# A new human-based metahurestic optimization method based on mimicking cooking training

**DOI:** 10.1038/s41598-022-19313-2

**Published:** 2022-09-01

**Authors:** Eva Trojovská, Mohammad Dehghani

**Affiliations:** Department of Mathematics, Faculty of Science, University of Hradec Králové, Rokitanského 62, Hradec Králové, 500 03 Czech Republic

**Keywords:** Electrical and electronic engineering, Mechanical engineering, Applied mathematics, Computer science

## Abstract

Metaheuristic algorithms have a wide range of applications in handling optimization problems. In this study, a new metaheuristic algorithm, called the chef-based optimization algorithm (CBOA), is developed. The fundamental inspiration employed in CBOA design is the process of learning cooking skills in training courses. The stages of the cooking training process in various phases are mathematically modeled with the aim of increasing the ability of global search in exploration and the ability of local search in exploitation. A collection of 52 standard objective functions is utilized to assess the CBOA’s performance in addressing optimization issues. The optimization results show that the CBOA is capable of providing acceptable solutions by creating a balance between exploration and exploitation and is highly efficient in the treatment of optimization problems. In addition, the CBOA’s effectiveness in dealing with real-world applications is tested on four engineering problems. Twelve well-known metaheuristic algorithms have been selected for comparison with the CBOA. The simulation results show that CBOA performs much better than competing algorithms and is more effective in solving optimization problems.

## Introduction

The technique of finding the best feasible solution among all existing ones is known as optimization. Optimization is used in designing and maintaining many engineering, economic, and even social systems to minimize the necessary costs or maximize profits. Due to the wide application of optimization in different sciences, this topic has grown a lot, so it is studied in management, mathematics, industry, and many branches of science^1^. If we want to solve a real optimization problem, we must first build the corresponding mathematical model. Setting up a model, of course, means creating a complete description of the problem with variables and mathematical relationships so that all the details of the optimization problem are simulated^2^.

Deterministic optimization methods can be divided into gradient-based and non-gradient methods, which effectively solve linear, convex, and derivable optimization problems and have a continuous search space. On the other hand, many real-world optimization problems have features such as nonlinear, non-convex objective functions, discrete search spaces, non-differentiable, high dimensions, and high complexity^3^.

The inability of deterministic methods to address such optimization challenges has led to the emergence of effective stochastic approaches in such cases. Metaheuristic algorithms, as the most prominent stochastic method, are capable of tackling optimization problems based on a random search, random operators, and trial-and-error processes^4^. The simplicity of concepts, easy implementation, efficiency in nonlinear and non-convex environments, and independence of the type of problem are the features that have led to the widespread use and popularity of metaheuristic algorithms^5^.

The primary source in the design of metaheuristic algorithms is inspiration from various natural phenomena, swarm intelligence, animal life, biological sciences, physical laws, rules of the game, and so on. Among the most famous metaheuristic algorithms are the genetic algorithm (GA)^6^, inspired by biology, the particle swarm optimization (PSO)^7^, the ant colony optimization (ACO)^8^, the Artificial bee colony (ABC)^9^, and the Northern Goshawk optimization^8^, inspired by animal life.

The critical issue with metaheuristic algorithms is that these methods do not guarantee that they will be able to find the optimal global solution. However, the solutions obtained from metaheuristic algorithms are close to the global optimal. The desire to achieve better solutions has led to the development of numerous metaheuristic algorithms.

Given the development of numerous metaheuristic algorithms, the main research question is, is there still a need to design newer algorithms? In answer to this question, the No Free Lunch (NFL) theorem^10^ states that the success of an algorithm in handling a set of optimization problems cannot be a reason for the successful performance of this algorithm in dealing with other optimization problems. There is no presumption of the success or failure of a method in optimizing a problem. The NFL theorem explains that no particular algorithm can be introduced as the best optimizer in all optimization applications. The NFL theorem is a source of motivation for researchers come up with better solutions to optimization problems by designing newer metaheuristic algorithms.

The innovation and novelty of the proposed chef-based optimization algorithm (CBOA) are:

- This paper introduces a new metaheuristic algorithm based on the description of a training process.
- Every educational process in different types of schools has certain usual properties, forms and stages. In this paper, we were concretely motivated by all specifics of the process of learning the cooking skills of a new chief.
- The paper provides a mathematical model of two-phase description of the preparation of a new chef, according to the principles of a real cooking school.
- Both phases are typical of all art schools (including cooking courses) because every student wants to learn from the best chef, but on the other hand, the greatest chefs will not want to prepare weak students. So, in the first phase, the chefs compete with each other so that a table of their quality ranking can be created. Similarly, in the second phase, students compete with each other so that their qualitative ranking can be created according to their cooking abilities.
- In the mathematical modeling of the first phase, we implemented two master chef strategies. These strategies model the fact that even chefs learn new cooking recipes by observing the teaching of other chefs (Strategy 1), and then they try to improve these observed recipes even more through their autonomous experimentation (Strategy 2).
- In the mathematical modeling of the first phase, we implemented three student strategies. The first strategy of each student is to choose a chef and learn all of his/her skills. The second strategy of each student is to choose another chef and learn from him/her one skill (one concrete recipe). In the third strategy, students try to improve all their skills by self-experimentation.
- CBOA ability to handle optimization problems is tested on fifty-two standard benchmark functions and compared with twelve well-known meta-heuristic algorithms. In doing so, CBOA achieves much better results than these competing programs.

The rest of the structure of the paper is as follows; the literature review is presented in the “Lecture review’’ section. The proposed CBOA is introduced and modeled in the “Chef-based optimization algorithm’’ section. The simulation studies and results are presented in the “Simulation studies and results’’ section. A discussion of results and performance of the proposed CBOA is presented in the “Discussion’’ section. CBOA implementation on CEC 2017 test suite is presented in “Evaluation CEC 2017 test suite” section. The efficiency of CBOA in handling real-world applications is evaluated in “CBOA for real world applications” section. Conclusions and several suggestions for future research are provided in the “Conclusions and future works’’ section.

## Lecture review

Metaheuristic algorithms, according to the primary source of design inspiration, are classified into five groups: (i) swarm-based, (ii) evolutionary-based, (iii) physics-based, (iv) game-based, and (v) human-based methods.

Theorizing on swarming activities and behaviors in the lives of birds, animals, aquatic animals, insects, and other living things in nature has been the main source of inspiration in the development of swarm-based algorithms. PSO, ACO, and ABC are among the most widely used and popular swarm-based algorithms. The natural behavior of the crowds of birds or fish in search of food have been the main idea of the PSO. Discovering the shortest path between the nest and the food source based on the collective intelligence of ants has been main idea of ACO. Hierarchical efforts and activities of bee colonies in search of food has been the main idea of the ABC. The idea of the ability of living organisms to find food sources in nature has led to the design of several swarm-based metaheuristic algorithms, such as: the tunicate swarm algorithm (TSA)^11^, the African vultures optimization algorithm (AVOA)^12^, and the snake optimizer (SO)^13^. The strategy of living things in nature when hunting and trapping prey has been the main idea in designing algorithms such as the grey wolf optimizer (GWO)^14^, the Golden Jackal optimization (GJO)^15^, the whale optimization algorithm (WOA)^16^, the reptile search algorithm (RSA)^17^, the marine predator algorithm (MPA)^18^.

The concepts of natural selection, Darwin’s theory of evolution, and stochastic operators such as selection, crossover, and mutation have been used in the design of evolutionary algorithms. GA and differential evolution (DE)^19^ are among the most famous evolutionary algorithms whose main design idea is the reproduction process and its concepts.

The laws, concepts, and phenomena of physics have been a source of inspiration in designing of numerous methods that fall into the category of physics-based algorithms. Simulated annealing (SA) is the most significant physics-based algorithm produced based on the physical phenomenon of metal annealing^20^. Physical forces and Newton’s laws of motion have been the main idea behind the design of methods such as the gravitational search algorithm (GSA) based on gravity force^21^ and the spring search algorithm (SSA) based on spring force^22^. Mathematical modeling of the natural water cycle in nature has led to the design of the water cycle algorithm (WCA)^23^. Cosmological studies and space holes have been the inspiration in designing the multi-verse optimizer (MVO)^24^. Archimedes principle concepts have been the main idea in the design of the archimedes optimization algorithm (AOA)^24^.

The rules of the game, the behavior of the players, the coaches, and the referees have been a source of inspiration for designing game-based algorithms. Football game based optimization (FGBO)^24^ and the volleyball premier league (VPL)^25^ are two game-based approaches designed based on the modeling of football and volleyball league, respectively. The strategy of the players to put the pieces together has been the design idea of the puzzle optimization algorithm (POA)^26^.

Human activities and behaviors in individual and social life have become the idea of designing approaches that fall into the category of human-based algorithms. Teaching–learning-based optimization (TLBO) is one of the most famous human-based algorithms that has been developed based on the simulation of interactions between a teacher and students in the classroom^27^. The treatment process that the doctor performs to treat patients has been the main idea in the design of the doctor and patient optimization (DPO)^28^. The cooperation of the members of a team to achieve success and the common goal of that team has been the main idea in the design of the teamwork optimization algorithm (TOA)^29^. The City Councils Evolution (CCE) is a human-based approach that is produced based on modeling the evolution of city councils^30^. The strategic movement of army troops during the war has been the idea employed in the design of the war strategy optimization (WSO)^31^.

Based on the best knowledge gained from the literature review, no metaheuristic algorithm inspired by the culinary education process has been designed. However, teaching cooking to people who attend training courses is an intelligent process that can be a motivation to design a new metaheuristic algorithm. Consequently, in this study, a new optimization approach has been developed by mathematical modeling the cooking education process, which is discussed in the next section.

### Ethical approval

This article does not contain any studies with human participants or animals performed by any of the authors.

### Informed consent

Informed consent was not required as no human or animals were involved.

## Chef-based optimization algorithm

This part is devoted to the introduction and mathematical modeling of the proposed algorithm called the Chef-based optimization algorithm (CBOA).

### Inspiration of CBOA

Cooking students and young cooks participate in training courses to improve their cooking skills and become chefs. This concept is analogous to metaheuristic algorithms, where several candidate solutions are initialized and then improved through an iterative process to determine the best candidate solution as the solution to the problem at the end of the algorithm implementation. Thus, the process of transforming a cooking student into a chef in a culinary school is a source of inspiration for the design of the proposed CBOA.

It is assumed that a certain number of chef instructors are present in a culinary school. Each chef instructor is responsible for teaching a class. Each cooking student can choose which of these classes to attend. The chef instructor teaches students cooking skills and techniques. However, chef instructors also try to improve their skills based on the instructions of the best chef instructor in the school and individual exercises. Cooking students try to learn and imitate the skills of the chef instructor. In addition, cooking students try to improve the skills they have learned through practice. At the end of the course, cooking students become skilled chefs under the training they have received.

Mathematical modeling of the above concepts is used in designing the CBOA, which is discussed in the following subsections.

### Algorithm initialization

The proposed CBOA approach is a population-based algorithm whose members consist of two groups of people, namely cooking students and chef instructors. Each CBOA member is a candidate solution that contains information about the problem variables. From a mathematical point of view, each member of the CBOA is a vector, and the set of CBOA members can be modeled using a matrix according to Eq. (1).

1 $$ X = \left[ {\begin{array}{*{20}c} {X_{1} } \\ \vdots \\ {X_{i} } \\ \vdots \\ {X_{N} } \\ \end{array} } \right]_{N \times m} = \left[ {\begin{array}{*{20}c} {x_{1,1} } & \cdots & {x_{1,j} } & \cdots & {x_{1,m} } \\ \vdots & \ddots & \vdots & {\mathinner{\mkern2mu\raise1pt\hbox{.}\mkern2mu \raise4pt\hbox{.}\mkern2mu\raise7pt\hbox{.}\mkern1mu}} & \vdots \\ {x_{i,1} } & \cdots & {x_{i,j} } & \cdots & {x_{i,m} } \\ \vdots & {\mathinner{\mkern2mu\raise1pt\hbox{.}\mkern2mu \raise4pt\hbox{.}\mkern2mu\raise7pt\hbox{.}\mkern1mu}} & \vdots & \ddots & \vdots \\ {x_{N,1} } & \cdots & {x_{N,j} } & \cdots & {x_{N,m} } \\ \end{array} } \right]_{N \times m} , $$

where $X$ is the CBOA population matrix, ${X}_{i}=\left({x}_{i,1},{x}_{i,2},\dots ,{x}_{i,m}\right)$ is the $i$th CBOA member (candidate solution), ${x}_{i,j}$ is its $j$th coordinate (i.e., the value of the $j$th problem variable for the $i$th CBOA member), $N$ is the population size, and $m$ is the number of problem variables of the objective function (dimension of the problem).

The position of the CBOA members at the beginning of the algorithm implementation is randomly initialized for $i=\text{1,2}, \dots , N \text{and} j=\text{1,2}, \dots ,m$ using Eq. (2).

2 $${x}_{i,j}=l{b}_{j}+r\cdot \left(u{b}_{j}-l{b}_{j}\right),$$

where $r$ is a random number in the interval $\left[\text{0,1}\right]$, $l{b}_{j}$ and $u{b}_{j}$ are the lower and the upper bounds of the $j$th problem variable, respectively.

By inserting the suggested values of each CBOA member into the variables, a corresponding objective function value is evaluated. As a result, the objective function is evaluated in $N$ turns (where $N$ is the number of CBOA members) and $N$ values are calculated for the objective function. These values can be represented using a vector corresponding to Eq. (3).

3 $$F={\left[\begin{array}{*{20}l}{F}_{1}\\ \vdots \\ {F}_{i}\\ \vdots \\ {F}_{N}\end{array}\right]}_{N\times 1}={\left[\begin{array}{*{20}l}F({X}_{1})\\ \vdots \\ F({X}_{i})\\ \vdots \\ F({X}_{N})\end{array}\right]}_{N\times 1},$$

where $F$ is the vector of values of the objective function and ${F}_{i}$ is the value of the objective function obtained for the $i$th member of CBOA, where $i=\text{1,2}, \dots , N.$

The values of the objective functions provide essential information about the quality of the candidate solutions. The value of the objective function is the decision criterion for selecting the best candidate solution. Among CBOA members, the member with the best value for the objective function is recognized as the best member of the population and the best candidate solution. During the running of the algorithm, in each iteration, the members of the CBOA are updated, and the corresponding values of the objective function are calculated. It is, therefore, necessary to update the best member in each iteration based on comparing the values of the objective function.

### Mathematical modeling of CBOA

After the algorithm is initialized, the CBOA steps are gradually applied to the candidate solutions to improve them. CBOA members consist of a group of instructing chefs and a group of cooking students. The update process for each of these groups is different. Based on comparing the values of the objective function, some CBOA members with better values of the objective function are selected as the chef instructor. Therefore, if the rows of the CBOA population matrix are sorted in ascending order according to the value of the objective function (thus, the member in the first row is the best member), then the group of the first ${N}_{C}$ members is selected as the group of chef instructors and the rest group of ${N-N}_{C}$ members is chosen as the group of cooking students. The CBOA sorted population matrix and the sorted objective function vector are specified in Eqs. (4) and (5).

4 $$ XS = \left[ {\begin{array}{*{20}c} {XS_{1} } \\ \vdots \\ {XS_{{N_{C} }} } \\ {XS_{{N_{C} + 1}} } \\ \vdots \\ {XS_{N} } \\ \end{array} } \right]_{N \times m} = \left[ {\begin{array}{*{20}c} {xs_{1,1} } & \cdots & {xs_{1,j} } & \cdots & {xs_{1,m} } \\ \vdots & \ddots & \vdots & {\mathinner{\mkern2mu\raise1pt\hbox{.}\mkern2mu \raise4pt\hbox{.}\mkern2mu\raise7pt\hbox{.}\mkern1mu}} & \vdots \\ {xs_{{N_{C} ,1}} } & \cdots & {xs_{{N_{C} ,j}} } & \cdots & {xs_{{N_{C} ,m}} } \\ {xs_{{N_{C} + 1,1}} } & \cdots & {xs_{{N_{C} + 1,j}} } & \cdots & {xs_{{N_{C} + 1,m}} } \\ \vdots & {\mathinner{\mkern2mu\raise1pt\hbox{.}\mkern2mu \raise4pt\hbox{.}\mkern2mu\raise7pt\hbox{.}\mkern1mu}} & \vdots & \ddots & \vdots \\ {xs_{N,1} } & \cdots & {xs_{N,j} } & \cdots & {xs_{N,m} } \\ \end{array} } \right]_{N \times m} , $$

5 $$FS={\left[\begin{array}{*{20}c}{FS}_{1}\\ \vdots \\ F{S}_{{N}_{C}}\\ F{S}_{{N}_{C}+1}\\ \vdots \\ {FS}_{N}\end{array}\right]}_{N\times m},$$

where ${N}_{C}$ is the number of chef instructors, $XS$ is the sorted population matrix of CBOA, and $FS$ is a vector of ascending objective function values. In the matrix $XS$, members from ${XS}_{1}$ to $X{S}_{{N}_{C}}$ represent the group of chef instructors, and members from $X{S}_{{N}_{C}+1}$ to $X{S}_{N}$ represent the group of cooking students. The vector $FS$ i includes successively the values of the objective functions corresponding to ${XS}_{1}$ to $X{S}_{N}$.

### Phase 1: the updating process for group of chef instructors (update of ${XS}_{1}$ to $X{S}_{{N}_{C}}$)

In a culinary school, it is assumed that several chef instructors are responsible for teaching cooking skills to students. Chef instructors follow two strategies to improve their cooking skills. In the first strategy, they emulate the best chef instructor and try to learn the chef instructor techniques. This strategy demonstrates the global search and CBOA exploration capabilities.

The advantage of updating the chef instructors based on this strategy is that the top chefs (top population members) improve their skills based on the best chef (best population member) before they start teaching students. Hence, there is no direct dependence on updating the students’ position only on the base of the best member of the population in CBOA design. Furthermore, this approach prevents the algorithm from getting stuck in local optima and causes different areas of the search space to be scanned more accurately and effectively. Based on this strategy, a new position for each chef instructor is first calculated for $i=\text{1,2}, \dots , {N}_{C} \,\text{and}\, j=\text{1,2}, \dots ,m$ using the following equation

6 $${xs}_{i,j}^{C/S1}={xs}_{i,j}+r\cdot \left({BC}_{j}-I\cdot {xs}_{i,j}\right),$$

where ${XS}_{i}^{C/S1}$ is the new calculated status for the $i$th sorted member of CBOA (that is ${XS}_{i}$) based on the first strategy ($C/S1$) of updating the chef instructor, ${xs}_{i,j}^{C/S1}$ is its $j$th coordinate, $BC$ is the best chef instructor (denoted as ${XS}_{1}$ in the matrix $XS$), ${BC}_{j}$ is the $j$th coordinate of the best chef instructor, $r$ is a random number from the interval $\left[\text{0,1}\right]$, and $I$ is a number that is selected randomly during execution from the set $\left\{\text{1,2}\right\}$. This new position is acceptable to the CBOA if it improves the value of the objective function. This condition is modeled using Eq. (7).

7 $${XS}_{i}=\left\{\begin{array}{*{20}l}{XS}_{i}^{C/S1}, & {FS}_{i}^{C/S1}<{F}_{i};\\ {XS}_{i}, & else,\end{array}\right.$$

where ${FS}_{i}^{C/S1}$ is the value of the objective function of the member ${XS}_{i}^{C/S1}.$

In the second strategy, each chef instructor tries to improve his cooking skills based on individual activities and exercises. This strategy represents the local search and the CBOA’s exploitation ability. If each problem variable is considered a cooking skill, a chef instructor will try to improve all of those skills to achieve a better objective function value.

The advantage of updating based on individual activities and exercises is that each member, regardless of the position of other population members, seeks to discover better solutions near the position where it is located. There is a possibility that better solutions can be obtained based on local search and exploitation, with minor changes in the position of population members in the search space. According to this concept, around each chef instructor in the search space, a random position is generated for $j=\text{1,2}, \dots ,m$ using Eqs. (8) to (10). If this random position improves the value of the objective function, it is acceptable for updating, which this condition is modeled using Eq. (11).

8 $$l{b}_{j}^{local}=\frac{l{b}_{j}}{t} ,$$

9 $$u{b}_{j}^{local}=\frac{u{b}_{j}}{t} ,$$

where $l{b}_{j}^{local}$ and $u{b}_{j}^{local}$ are the lower and upper local bound of the $j$th problem variable, respectively, and the variable $t$ represents the iteration counter.

10 $${xs}_{i,j}^{C/S2}={xs}_{i,j}+l{b}_{j}^{local}+r\cdot \left(u{b}_{j}^{local}-l{b}_{j}^{local}\right), i=\text{1,2}, \dots , {N}_{C}, j=\text{1,2}, \dots ,m,$$

11 $${XS}_{i}=\left\{\begin{array}{*{20}l}{XS}_{i}^{C/S2}, & {FS}_{i}^{C/S2}<{F}_{i};\\ {XS}_{i}, & else,\end{array}\right.$$

where ${XS}_{i}^{C/S2}$ is the new calculated status for the $i$th CBOA sorted member (i.e., ${XS}_{i}$) based on the second strategy ($C/S2$) of chef instructors updating, ${xs}_{i,j}^{C/S2}$ is its $j$th coordinate, and ${FS}_{i}^{C/S2}$ is its value of the objective function.

### Phase 2: the updating process for the group of cooking students (update of ${XS}_{{N}_{C}+1}$ to $X{S}_{N}$)

Cooking students attend culinary school to learn cooking skills and become a chef. In the design of CBOA, it is assumed that cooking students follow three strategies to learn cooking skills. According to the first strategy, each cooking student randomly chooses a class taught by one of the chefs, and then he is taught cooking skills by this chef instructor. The advantage of updating cooking students based on this strategy is that there are different chef instructors available to lead them, resulting in cooking students learning different skills (i.e., population members moving to other areas of the search space) based on the guidance of the chosen chef instructor. On the other hand, if all cooking students learn only from the best chef-instructor (all members of the population moved towards the best member), then an efficient global search in the problem-solving space would not be possible. This strategy is simulated in the CBOA in such a way that first for each cooking student, a new position is calculated based on the training and guidance of the chef instructor, for $i={N}_{C}+1, {N}_{C}+2, \dots , N, j=\text{1,2}, \dots ,m,$ using Eq. (12).

12 $${xs}_{i,j}^{S/S1}={xs}_{i,j}+r\cdot \left({CI}_{{k}_{i},j}-I\cdot {xs}_{i,j}\right),$$

where ${XS}_{i}^{S/S1}$ is the new calculated status for the $i$th sorted member of CBOA (i.e., ${XS}_{i}$) based on the first strategy ($S/S1$) of the updating of cooking students, ${xs}_{i,j}^{S/S1}$ is its $j$th coordinate, and ${CI}_{{k}_{i},j}$ is the selected chef instructor by the $i$th cooking student, where ${k}_{i}$ is randomly selected from the set $\left\{\text{1,2}, \dots , {N}_{C}\right\}$ (where ${CI}_{{k}_{i},j}$ denotes the value ${xs}_{{k}_{i},j}$).

This new position replaces the previous position for each CBOA member, if it improves the value of the objective function. This concept is modeled for $i={N}_{C}+1, {N}_{C}+2, \dots , N$ by Eq. (13).

13 $${XS}_{i}=\left\{\begin{array}{*{20}l}{XS}_{i}^{S/S1}, & {FS}_{i}^{S/S1}<{F}_{i};\\ {XS}_{i}, & else,\end{array}\right.$$

where ${FS}_{i}^{S/S1}$ is the value of the objective function of ${XS}_{i}^{S/S1}.$

In the second strategy, since each problem variable in the CBOA is assumed to be a cooking skill, each cooking student tries to learn one of the skills of the chef instructor completely and fully imitate the chef instructor (therefore, by “skill’’, we mean a recipe for one great meal). This strategy enhances the global search and exploration capabilities of the CBOA. The advantage of this strategy is that instead of updating all candidate solution variables (i.e., all cooking student skills), only one variable (one skill, i.e., one recipe) changes. It may not be necessary to update all member position coordinates to achieve better solutions.

In the design of CBOA, this “skill’’ represents a certain component of a vector of cooking skills of a randomly selected chef instructor ${CI}_{k}$ ($k\in \left\{\text{1,2},\dots ,{N}_{c}\right\}$). Hence, the second strategy is mathematically simulated in such a way that for each cooking student ${XS}_{i}$ (members of CBOA with $i={N}_{C}+1, {N}_{C}+2, \dots , N$), first one chief instructor, which is represented by the vector ${CI}_{{k}_{i}}=\left({CI}_{{k}_{i,1}}, \dots , {CI}_{{k}_{i,m}}\right),$ is randomly selected (a member of CBOA with the index ${k}_{i}$, which is randomly selected from the set $\{1,..., {N}_{C}\}$), then it is randomly selected his $\mathcal{l}$th coordinate (thus a number $\mathcal{l}$ from the set $\left\{1,... m\right\},$ which represents a “skill’’ of this selected chief instructor) and by this value ${CI}_{{k}_{i,\mathcal{l}}}$ we replace the $\mathcal{l}$th coordinate of the vector of the $i$th cooking student ${XS}_{i}$ (thus, ${xs}_{i,\mathcal{l}}$).

According to this concept, a new position is calculated for each CBOA cooking student member using Eq. (14).

14 $${xs}_{i,j}^{S/S2}=\left\{\begin{array}{*{20}l}{CI}_{{k}_{i},j}, & j=l;\\ {xs}_{i,j}, & else,\end{array}\right.$$

where $\mathcal{l}$ is a randomly selected number from the set $\left\{\text{1,2}, \dots ,m\right\},$ $i={N}_{C}+1, {N}_{C}+2, \dots , N,$ $j=\text{1,2}, \dots ,m.$ Then, it is replaced with the previous position based on Eq. (15) if it improves the target value of the objective function.

15 $${XS}_{i}=\left\{\begin{array}{*{20}l}{XS}_{i}^{S/S2}, & {FS}_{i}^{S/S2}<{F}_{i};\\ {XS}_{i}, & else,\end{array}\right.$$

where ${XS}_{i}^{S/S2}$ is the new calculated status for the $i$th sorted member of CBOA (i.e., ${XS}_{i}$) based on the second strategy ($S/S2$) of updating cooking students, ${xs}_{i,j}^{S/S2}$ is its $j$th coordinate, ${FS}_{i}^{S/S2}$ is its objective function value.

In the third strategy, each cooking student tries to improve his cooking skills based on his individual activities and exercises. In fact, this strategy represents the local search and the CBOA’s exploitation ability. The advantage of updating cooking students based on the strategy of individual activities and exercises is that it increases the power of local search and exploitation of the algorithm in achieving better possible solutions near the discovered solutions. In this strategy, similar to the local search strategy of chef instructors, cooking students try to converge to better solutions with small and precise steps. If each problem variable is considered a cooking skill, a cooking student will try to improve all of those skills to achieve a better objective function value.

According to this concept, around each cooking student in the search space, a random position is generated by Eqs. (8), and (9) and a new position is calculated using Eq. (16).

16 $${xs}_{i,j}^{S/S3}=\left\{\begin{array}{*{20}l}{xs}_{i,j}+l{b}_{j}^{local}+r\cdot \left(u{b}_{j}^{local}-l{b}_{j}^{local}\right), & j=q; \\ {xs}_{i,j}, & j\ne q,\end{array}\right.$$

where ${XS}_{i}^{S/S3}$ is the new calculated status for the $i$th sorted member of CBOA (that is ${XS}_{i}$) based on the third strategy ($S/S3$) of updating cooking students, ${xs}_{i,j}^{S/S3}$ is its $j$th coordinate, and $q$ is randomly selected number from the set $\left\{\text{1,2}, \dots ,m\right\}$, $i={N}_{C}+1, {N}_{C}+2, \dots , N$, and $j=\text{1,2}, \dots ,m.$ If this new random position improves the value of the objective function, it is acceptable for updating of ${XS}_{i}$, which is modeled by Eq. (17).

17 $${XS}_{i}=\left\{\begin{array}{*{20}l}{XS}_{i}^{S/S3}, & {FS}_{i}^{S/S3}<{F}_{i};\\ {XS}_{i}, & else,\end{array}\right.$$

where ${FS}_{i}^{S/S3}$ is the value of the objective function of ${XS}_{i}^{S/S3}.$

### Repetition process, pseudocode, and flowchart of CBOA

**Figure 1:**
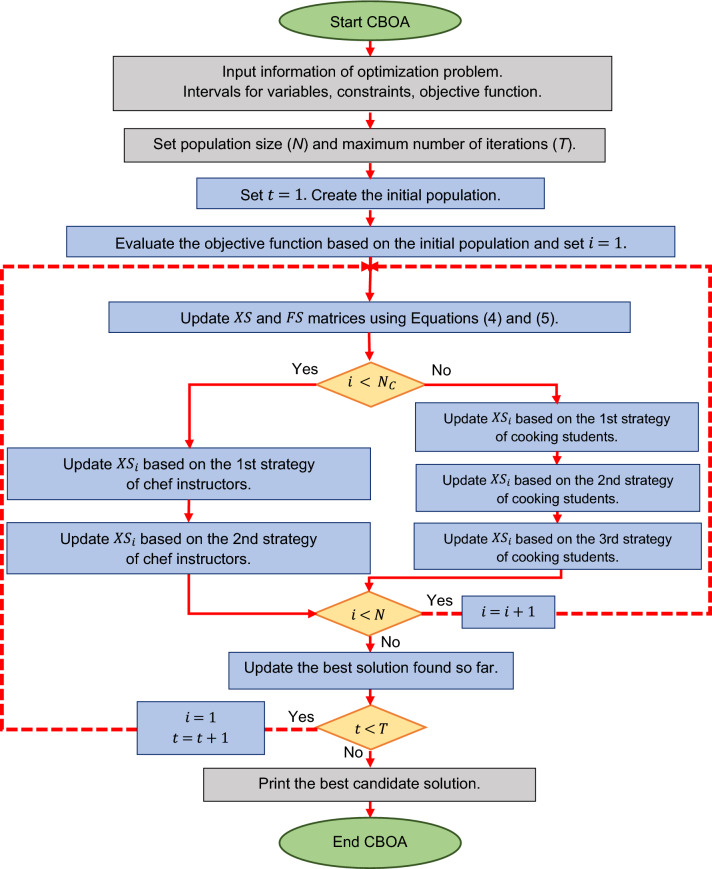
Flowchart of CBOA.


A CBOA iteration is completed by updating all members of the population. The CBOA enters the next iteration with these new statuses, and the groups of chef instructors and cooking students are respecified. The population members are updated based on the implementation of the CBOA steps according to Eqs. (4) to (17) until the last iteration of the algorithm. After reaching the maximum value of the iteration variable CBOA, the best candidate solution obtained during the implementation process is presented as the solution to the problem. Various steps of CBOA implementation are presented in the form of a flowchart in Fig. 1 and its pseudocode in Algorithm 1.

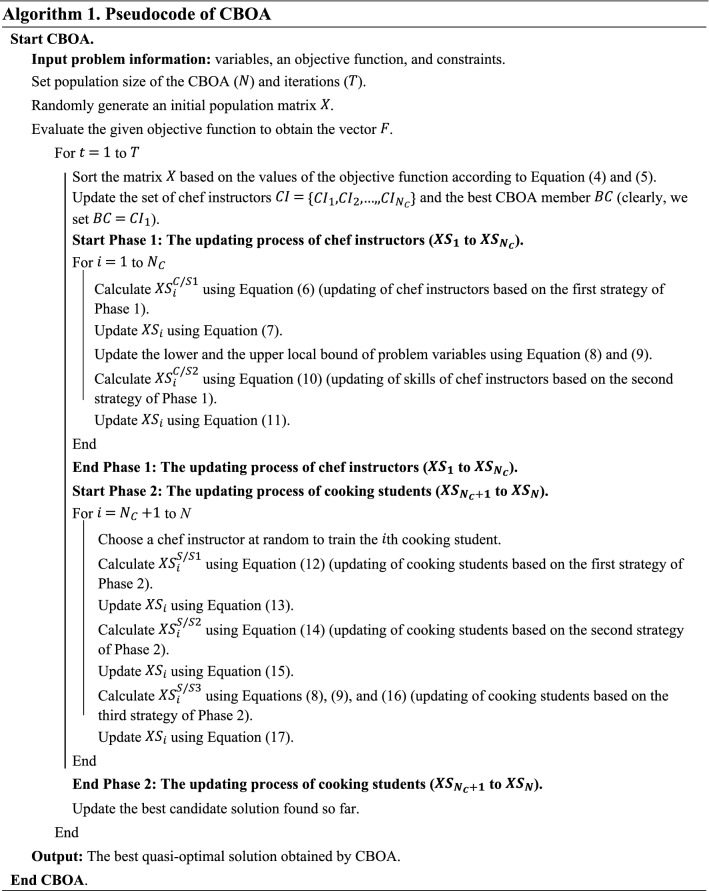


### Computational complexity of CBOA

In this subsection, the computational complexity of the CBOA is analyzed. Preparing and initializing the CBOA for an optimization problem, with the number of decision variables $m$, has a computational complexity of $O(Nm)$, where $N$ is the number of CBOA members. Updating the group of chef instructors in two strategies has a computational complexity equal to $O(2{N}_{C}mT)$, where *T* is the maximum number of CBOA iterations and ${N}_{C}$ is the number of chef instructors. Updating the student cooking group in three strategies has a computational complexity equal to $O(3 (N-{N}_{C}) mT)$. Thus, the total computational complexity of CBOA is equal to $O(m(N + 2{N}_{C}T + 3 \left(N-{N}_{C}\right)T ))$.

## Simulation studies and results

This section presents simulation studies and an evaluation of the ability of CBOA to solve optimization problems and real practice tasks. For this purpose, a set of 23 standard benchmark objective functions has been employed. For this purpose, a set of 23 standard benchmark objective functions has been employed. The reason for choosing this collection is as follows. Seven unimodal functions ${F}_{1}$ to ${F}_{7}$, which have only one main extremum and lack local optimal solutions, have been selected. Therefore, unimodal functions are employed to challenge the exploitation and local search ability of the proposed CBOA algorithm in convergence to global optimal. The six functions in this set, ${F}_{8}$ to ${F}_{13}$, are the high-dimensional multimodal type, which, in addition to the main extremum, has several local extremums and local optimal solutions. Thus, high-dimensional multimodal functions are employed to test the CBOA’s exploration and global search capability in accurately scanning the search space, passing local optimal areas, and discovering the main optimal area. The ten functions in this set, ${F}_{14}$ to ${F}_{23}$, are selected from the fixed-dimensional multimodal type, whose dimensions and the number of local extremes are less than those of the high-dimensional multimodal functions. These functions are employed to analyze the ability of the proposed CBOA algorithm to strike a balance between exploration and exploitation. The information on this set of benchmark functions is specified in Tables 1, 2 and 3.

**Table 1:** Information about unimodal objective functions.

Objective function		Range	Dimensions ($m$)	${F}_{min}$
1.	${F}_{1}\left(x\right)={\sum }_{i=1}^{m}{x}_{i}^{2}$	${\left[-\,100, 100\right]}^{m}$	30	0
2.	${F}_{2}\left(x\right)={\sum }_{i=1}^{m}\left|{x}_{i}\right|+ {\prod }_{i=1}^{m}\left|{x}_{i}\right|$	${\left[-\,10, 10\right]}^{m}$	30	0
3.	${F}_{3}\left(x\right)={\sum }_{i=1}^{m}{\left({\sum }_{j=1}^{i}{x}_{i}\right)}^{2}$	${\left[-\,100, 100\right]}^{m}$	30	0
4.	${F}_{4}\left(x\right)=\text{max}\left\{\left|{x}_{i}\right| , 1\le i\le m\right\}$	${\left[-\,100, 100\right]}^{m}$	30	0
5.	${F}_{5}\left(x\right)={\sum }_{i=1}^{m-1}\left(100{\left({x}_{i+1}-{x}_{i}^{2}\right)}^{2}+{\left({x}_{i}-1\right)}^{2})\right)$	${\left[-\,30, 30\right]}^{m}$	30	0
6.	$F_{6} \left( x \right) = \mathop \sum \limits_{i = 1}^{m} \left\lfloor {x_{i} + 0.5} \right\rfloor^{2}$	${\left[-\,100, 100\right]}^{m}$	30	0
7.	${F}_{7}\left(x\right)={\sum }_{i=1}^{m}{ix}_{i}^{4}+random(\text{0,1}]$	${\left[-\,1.28, 1.28\right]}^{m}$	30	0

**Table 2:** Information about high-dimensional multimodal objective functions.

Objective function		Range	Dimensions ($m$)	${F}_{min}$
8.	${F}_{8}\left(x\right)={\sum }_{i=1}^{m}-{x}_{i}\text{sin}(\sqrt{|{x}_{i}|})$	${\left[-\,500, 500\right]}^{m}$	30	$- \,418.98 m$
9.	${F}_{9}\left(x\right)={\sum }_{i=1}^{m}\left({x}_{i}^{2}-10\text{cos}\left(2\pi {x}_{i}\right)+10\right)$	${\left[-\,5.12, 5.12\right]}^{m}$	30	0
10.	${F}_{10}\left(x\right)=-20\text{exp}\left(-0.2\sqrt{\frac{1}{m}{\sum }_{i=1}^{m}{x}_{i}^{2}}\right)$ $-\text{exp}\left(\frac{1}{m}{\sum }_{i=1}^{m}\text{cos }\left(2\pi {x}_{i}\right)\right)+20+e$	${\left[-\, 32, 32\right]}^{m}$	30	0
11.	${F}_{11}\left(x\right)=\frac{1}{4000}{\sum }_{i=1}^{m}{x}_{i}^{2}- {\prod }_{i=1}^{m}\text{cos}\left(\frac{{x}_{i}}{\sqrt{i}}\right)+1$	${\left[-\,600, 600\right]}^{m}$	30	0
12.	${F}_{12}\left(x\right)=\frac{\pi }{m} \{10\text{sin}\left(\pi {y}_{1}\right)$ $+{\sum }_{i=1}^{m-1}{\left({y}_{i}-1\right)}^{2}\left(1+10{\text{sin}}^{2}\left(\pi {y}_{i+1}\right)\right)+{\left({y}_{m}-1\right)}^{2}\}+{\sum }_{i=1}^{m}u\left({x}_{i},\text{10,100,4}\right),$ where ${y}_{i}=1+\frac{1+{x}_{i}}{4}$, $$u\left({x}_{i},a,i,n\right)= \left\{\begin{array}{*{20}l}k{\left({x}_{i}-a\right)}^{n}, {x}_{i}>-a;\\ 0, -a\le {x}_{i} \le a\\ k{\left({-x}_{i}-a\right)}^{n}, {x}_{i}<-a.\end{array}\right.;$$	${\left[-\,50, 50\right]}^{m}$	30	0
13.	${F}_{13}\left(x\right)=0.1 \{ {\text{sin}}^{2}\left(3\pi {x}_{1}\right)+ {\sum }_{i=1}^{m}{\left({x}_{i}-1\right)}^{2}\left[1+{\text{sin}}^{2}\left(3\pi {x}_{i}+1\right)\right]+{\left({x}_{n}-1\right)}^{2}\left[1+{\text{sin}}^{2}\left(2\pi {x}_{m}\right)\right]\}+{\sum }_{i=1}^{m}u({x}_{i}, 5, 100, 4)$	${\left[-\,50, 50\right]}^{m}$	30	0

**Table 3:** Information about fixed-dimensional multimodal objective functions.

Objective function		Range	Dimensions ($m$)	${F}_{min}$
14.	${F}_{14}\left(x\right)={\left(\frac{1}{500}+{\sum }_{j=1}^{25}\frac{1}{j+{\sum }_{i=1}^{2}{\left({x}_{i}-{a}_{ij}\right)}^{6}}\right)}^{-1}$	${\left[-\, 65.53, 65.53\right]}^{2}$	$2$	$0.998$
15.	${F}_{15}\left(x\right)={\sum }_{i=1}^{11}{\left[{a}_{i}-\frac{{x}_{1}({b}_{i}^{2}+{b}_{i}{x}_{2})}{{b}_{i}^{2}+{b}_{i}{x}_{3}+{x}_{4}}\right]}^{2}$	${\left[-\, 5, 5\right]}^{4}$	$4$	$0.00030$
16.	${F}_{16}\left(x\right)=4{x}_{1}^{2}-2.1{x}_{1}^{4}+\frac{1}{3}{x}_{1}^{6}+{x}_{1}{x}_{2}-4{x}_{2}^{2}+4{x}_{2}^{4}$	${\left[- \,5, 5\right]}^{2}$	$2$	$- \,1.0316$
17.	${F}_{17}\left(x\right)={\left({x}_{2}-\frac{5.1}{4{\pi }^{2}}{x}_{1}^{2}+\frac{5}{\pi }{x}_{1}-6\right)}^{2}+$ $10\left(1-\frac{1}{8\pi }\right)\text{cos}{x}_{1}+10$	[− 5, 10]$\times$[0, 15]	$2$	$0.398$
18.	${F}_{18}\left(x\right)=[1$ $+{\left({x}_{1}+{x}_{2}+1\right)}^{2}\left(19-14({x}_{1}-{x}_{2})+{\left({x}_{1}+{x}_{2}\right)}^{2}\right)]\cdot [30+{\left(2{x}_{1}-3{x}_{2}\right)}^{2}\cdot (18-32{x}_{1}+12{x}_{1}^{2}+48{x}_{2}-36{x}_{1}{x}_{2}+27{x}_{2}^{2})]$	${\left[-\, 5, 5\right]}^{2}$	$2$	$3$
19.	${F}_{19}\left(x\right)=-{\sum }_{i=1}^{4}{c}_{i}\text{exp}(-{\sum }_{j=1}^{3}{a}_{ij}{\left({x}_{j}-{P}_{ij}\right)}^{2})$	${\left[0, 1\right]}^{3}$	$3$	$- \,3.86$
20.	${F}_{20}\left(x\right)=-{\sum }_{i=1}^{4}{c}_{i}\text{exp}(-{\sum }_{j=1}^{6}{a}_{ij}{\left({x}_{j}-{P}_{ij}\right)}^{2})$	${\left[0, 1\right]}^{6}$	$6$	$-\, \,3.32$
21.	${F}_{21}\left(x\right)=-{\sum }_{i=1}^{5}{\left[\left(X-{a}_{i}\right)\cdot {\left(X-{a}_{i}\right)}^{T}+6{c}_{i}\right]}^{-1}$	${\left[0, 10\right]}^{4}$	$4$	$-\,10.1532$
22.	${F}_{22}\left(x\right)=-{\sum }_{i=1}^{7}{\left[\left(X-{a}_{i}\right)\cdot {\left(X-{a}_{i}\right)}^{T}+6{c}_{i}\right]}^{-1}$	${\left[0, 10\right]}^{4}$	$4$	$-\,10.4029$
23.	${F}_{23}\left(x\right)=-{\sum }_{i=1}^{10}{\left[\left(X-{a}_{i}\right)\cdot {\left(X-{a}_{i}\right)}^{T}+6{c}_{i}\right]}^{-1}$	${\left[0, 10\right]}^{4}$	$4$	$-\,10.5364$

The performance of the proposed CBOA approach in optimization is compared with the results of 12 well-known metaheuristic algorithms. The criterion for selecting these 12 competitor algorithms is as follows. PSO, GA, and DE are three prevalent algorithms that have been employed in many optimization applications. CMA, GSA, TLBO, GWO, MVO, and WOA are the six most cited algorithms that always have interested researchers. Finally, the three algorithms, MPA, TSA, and HBO, are the algorithms that have been released recently and have received a lot of attention and application in this short period. The values adopted for the control parameters of the competitor algorithms are specified in Table 4.

**Table 4:** Adopted values for control parameters of competitor metaheuristic algorithms.

Algorithm	Parameter	Value
GA	Population size	100
	Type	Real coded
	Selection	Roulette wheel (Proportionate)
	Crossover	Whole arithmetic ($\text{Probability} = 0.8$, $\alpha \in \left[-\, 0.5, 1.5\right]$)
	Mutation	Gaussian (Probability = 0.05)
PSO	Population size	50
	Topology	Fully connected
	Cognitive and social constant	$\left({C}_{1}, {C}_{2}\right)= (2, 2),$
	Inertia weight	Linear reduction from 0.9 to 0.1
	Velocity limit	10% of the dimension range
GSA	Population size	50
	Alpha, *G*_*0*_, *R*_*norm*_, *R*_*power*_	20, 100, 2, 1
TLBO	Population size	50
	${T}_{F}$: teaching factor	${T}_{F}=\text{ round} (1+rand),$ where
	random number	*rand* is a random number in $\left[0, 1\right]$
GWO	Population size	30
	Convergence parameter (*a*)	*a*: Linear reduction from 2 to 0
MVO	Population size	30
	Wormhole existence probability (WEP)	$\text{Min}(WEP)=0.2$ and $\text{Max}(WEP)=1$
	Exploitation accuracy over iterations (*p*)	$p = 6$
WOA	Population size	30
	Convergence parameter (*a*)	*a*: Linear reduction from 2 to 0
	*r* is a random vector in the interval $\left[0, 1\right]$	
	*l* is a random number in $\left[-\,1, 1\right]$	
TSA	Population size	30
	${P}_{min}$ and ${P}_{max}$	1, 4
	${c}_{1}, {c}_{2}, {c}_{3}$	Random numbers from the interval $\left[0, 1\right]$
MPA	Population size	30
	Constant number	$P=0.5$
	Random vector	$R$ is a vector of uniform random numbers from the interval $\left[0, 1\right]$
	Fish aggregating devices (*FADs*)	$FADs=0.2$
	Binary vector	$U= 0$ or 1
HBA	Population size	30
	The ability of a honey badger to get food	$\beta =6$
	Constant number	$C=2$
DE	Population size	100
	Scaling factor	0.5
	Crossover probability	0.5
CMA	Num taps	5
	Step size	0.05
	Leakage factor	1
CBOA	Population size	30

The CBOA and each of the competing algorithms are tested on benchmark functions in twenty independent implementations while each execution contains 1000 iterations. Optimization results are reported using six indicators: mean, best, standard deviation (std), median, execution time (ET), and rank.

The CBOA and each competing algorithm are tested on benchmark functions in twenty independent implementations, while each execution contains 1000 iterations. Optimization results are reported using six indicators: mean, best, standard deviation (std), median, execution time (ET), and rank.

### Evaluation unimodal objective function

The optimization results of the unimodal functions ${F}_{1}$ to ${F}_{7}$ using CBOA and competitor algorithms are given in Table 5. The optimization results show that the CBOA has performed very well in optimizing ${F}_{1}$, ${F}_{2}$, ${F}_{3}$, ${F}_{4}$, and ${F}_{6}$ and has been able to converge to the global optimal of these functions. In optimizing the functions ${F}_{5}$ and ${F}_{7}$, the CBOA has been able to deliver good results and rank the best optimizer among the compared algorithms. The simulation results show that CBOA has a self-evident superiority over competitor algorithms and, with high exploitation ability, has converged to very suitable solutions.

**Table 5:** Results of optimization of CBOA and competitor metaheuristics on the unimodal function.

		CBOA	CMA	DE	HBA	MPA	TSA	WOA	MVO	GWO	TLBO	GSA	PSO	GA
${F}_{1}$	Mean	0	3.87E−12	2.65E−12	4.4E−277	2.95E−50	1.41E−46	2.5E−154	0.165132	8.49E−59	9.24E−74	1.06E−16	0.168284	32.30955
	Best	0	1.08E−12	1.09E−12	2.4E−287	1.55E−52	2.63E−51	1E−164	0.098704	3.66E−61	6.41E−77	4.4E−17	2.32E−06	17.01188
	Std	0	1.8E−12	1.13E−12	0	4.89E−50	4.47E−46	1E−153	0.044016	1.53E−58	3.28E−73	5.98E−17	0.377572	9.537409
	Median	0	3.55E−12	2.25E−12	1.8E−279	6.52E−51	5E−48	2.1E−157	0.157209	2.47E−59	8.67E−75	8.71E−17	0.002097	29.40147
	ET	2.172735	6.092415	5.748222	0.553321	3.445383	1.148152	0.568891	3.074551	1.93628	1.905436	3.862345	0.537751	0.815548
	Rank	1	10	9	2	6	7	3	11	5	4	8	12	13
${F}_{2}$	Mean	0	7E−06	3.25E−08	4.4E−146	8.73E−28	6.93E−29	7.1E−106	0.267917	1.08E−34	6.48E−39	8.26E−08	1.186001	2.812708
	Best	0	3.82E−06	1.65E−08	5E−150	3.2E−30	7.49E−30	7.4E−115	0.137216	1.36E−35	8.26E−40	4.05E−08	0.141104	1.815701
	Std	0	2.94E−06	8.52E−09	1.5E−145	1.65E−27	8.97E−29	1.8E−105	0.057929	8.92E−35	8.58E−39	1.18E−07	2.235033	0.477151
	Median	0	5.8E−06	3.11E−08	7.1E−148	1.95E−28	3.51E−29	6.1E−108	0.261335	8.56E−35	3.94E−39	5.22E−08	0.664504	2.832172
	ET	2.219213	6.066346	5.848163	0.564548	3.160192	1.175819	0.597698	2.799057	2.012479	2.011396	3.873261	0.531398	0.779758
	Rank	1	10	8	2	7	6	3	11	5	4	9	12	13
${F}_{3}$	Mean	0	6.83E−05	24,716.62	7.9E−204	9.31E−13	1.9E−11	15,520.85	13.69663	8.79E−16	3.76E−25	479.0439	898.3863	2271.434
	Best	0	1.15E−05	18,432.73	9.2E−220	4.38E−17	1.83E−19	3015.781	7.076154	3.09E−19	4.18E−29	214.1529	46.09636	1199.627
	Std	0	5.08E−05	3946.206	0	1.73E−12	7.11E−11	9780.884	5.317871	1.73E−−15	7.87E−25	140.3307	1516.41	827.2212
	Median	0	5.93E−05	24,419.55	1.6E−208	2.79E−14	2.97E−14	12,635.08	12.11916	1.09E−16	1.98E−26	463.5805	502.566	2112.874
	ET	6.621329	7.408674	7.488921	2.055253	7.210372	2.876052	2.174281	6.278508	3.558108	6.204809	5.127676	1.936225	2.226537
	Rank	1	7	13	2	5	6	12	8	4	3	9	10	11
${F}_{4}$	Mean	0	0.000159	1.983901	9.5E−120	3.17E−19	0.009342	28.50847	0.500893	1.71E−14	3E−30	1.345517	6.592888	3.143331
	Best	0	8.69E−05	1.316701	3.5E−124	4.5E−20	0.000147	0.001866	0.247351	1.28E−15	6.86E−32	1.54E−08	4.131017	1.999125
	Std	0	5.52E−05	0.343872	1.9E−119	2.31E−19	0.012302	31.87155	0.135805	2.66E−14	3.42E−30	1.598979	2.599888	0.582495
	Med	0	0.000151	2.000134	3.7E−121	2.92E−19	0.002038	16.99128	0.51331	8.78E−15	1.55E−30	0.805803	6.449158	3.157808
	ET	2.134919	6.048563	5.446143	0.553277	3.030702	1.113022	0.564871	2.946676	1.425534	1.915917	3.81435	0.541684	0.748298
	Rank	1	6	10	2	4	7	13	8	5	3	9	12	11
${F}_{5}$	Mean	0.000306	62.99214	52.93904	21.83121	23.47877	28.18633	27.05095	336.8397	26.69749	27.00714	40.06591	113.1056	445.1666
	Best	7.21E−05	17.59206	26.21261	20.89173	22.33217	26.36048	26.42674	27.57318	25.25594	25.76632	25.87646	22.77282	231.7436
	Std	0.00023	142.2852	27.60379	0.511057	0.472798	0.82398	0.363525	649.711	0.669329	0.992995	53.68502	90.0744	177.5128
	Median	0.000232	19.38299	39.00547	21.94289	23.49991	28.63377	27.05451	45.72737	27.09079	26.51047	26.16945	86.01345	380.1141
	ET	2.910138	6.151182	5.975958	0.820889	3.802344	1.386446	0.876656	3.45164	1.849231	2.616349	544.2818	0.765122	0.998529
	Rank	1	10	9	2	3	7	6	12	4	5	8	11	13
${F}_{6}$	Mean	0	4.49E−12	2.65E−12	9.74E−08	1.6E−09	3.225523	0.094859	0.155856	0.65113	1.170598	1.04E−16	0.230787	31.80092
	Best	0	1.55E−12	5.64E−13	5.49E−09	8.41E−10	2.295798	0.003153	0.058846	2.06E−05	0.243967	4.72E−17	6.24E−05	17.06432
	Std	0	1.99E−12	1.2E−12	1.27E−07	7.43E−10	0.530484	0.118184	0.047567	0.436312	0.46836	3.05E−17	0.969372	14.38352
	Median	0	4.18E−12	2.88E−12	4.64E−08	1.48E−09	3.069241	0.050352	0.152637	0.621537	1.1358	1E−16	0.004883	26.29787
	ET	2.213583	6.045107	5.258846	0.629315	3.078155	1.132735	0.691941	2.934654	2.037816	2.064517	1555.628	0.566689	0.766043
	Rank	1	4	3	6	5	12	7	8	10	11	2	9	13
${F}_{7}$	Mean	4.26E−05	0.032899	0.027278	5.31E−05	0.000759	0.00571	0.001145	0.011238	0.000888	0.002197	0.058194	0.168787	0.008934
	Best	5.39E−06	0.017076	0.019679	3.7E−05	0.000128	0.001473	9.36E−06	0.007012	0.000149	0.000448	0.021831	0.078608	0.004354
	Std	2.38E−05	0.009295	0.004461	2.78E−05	0.000428	0.003007	0.001365	0.00353	0.000638	0.001353	0.021042	0.068875	0.002685
	Median	3.84E−05	0.029407	0.027521	4.44E−05	0.000718	0.004762	0.00054	0.010196	0.000704	0.002016	0.053531	0.14742	0.008549
	ET	4.444716	6.626775	5.918818	1.427673	4.863401	1.857502	1.622592	4.414998	2.362614	4.26488	4.553437	1.232753	1.46431
	Rank	1	11	10	2	3	7	5	9	4	6	12	13	8
Sum rank		7	58	62	18	33	52	49	67	37	36	57	79	82
Mean rank		1	8.285714	8.857143	2.5714285	4.714286	7.428571	7	9.571429	5.285714	5.142857	8.142857	11.28571	11.71429
Total rank		1	9	10	2	3	7	6	11	5	4	8	12	13

### Evaluation high-dimensional multimodal objective function

Results of CBOA and all competitor algorithms on high-dimensional multimodal functions of ${F}_{8}$ to ${F}_{13}$ are reported in Table 6. CBOA has achieved precisely the global optimal solution for ${F}_{9}$ and ${F}_{11}$, which shows us the high exploration power of CBOA. In optimizing the function ${F}_{10}$, the proposed CBOA has performed well, and for this function is ranked as the first best optimizer in competition with the compared algorithms. The simulation results indicate the high exploration power of CBOA in identifying the best optimal region and the superiority of CBOA compared to competitor algorithms.

**Table 6:** Results of optimization of CBOA and competitor metaheuristics on the high-dimensional multimodal function.

		CBOA	CMA	DE	HBA	MPA	TSA	WOA	MVO	GWO	TLBO	GSA	PSO	GA
${F}_{8}$	Mean	− 11,416.7	− 4,801,045	− 12,454.5	− 8544.1	− 9692.45	− 6130.76	− 9160.03	− 7682.93	− 6049.79	− 5508.84	− 2618.05	− 6579.01	− 8661.75
	Best	− 12,332.6	− 4.4E+07	− 12,569.5	− 10,097.4	− 10,570.1	− 7089.62	− 11,847.1	− 8717.73	− 7464.67	− 6946.93	− 3587.57	− 8062.67	− 9997.91
	Std	608.3322	10,233,996	124.9645	1197.938	453.6502	540.5303	1934.878	518.1982	718.0628	860.7038	541.5419	813.4314	727.7971
	Median	− 11,572.6	− 608,750	− 12,451	− 8477.34	− 9710.72	− 6223.05	− 8800.53	− 7736.1	− 5876.46	− 5527.42	− 2448.32	− 6554.02	− 8708.15
	ET	3.32474	6.272527	5.682899	0.9268	3.733725	1.363383	1.03715	2.590324	1.651692	3.011316	4.237375	0.816449	1.100977
	Rank	3	1	2	7	4	10	5	8	11	12	13	9	6
${F}_{9}$	Mean	0	44.71531	61.37856	0	0	176.2674	0	97.68891	1.42E−14	0	27.46085	60.91495	59.92033
	Best	0	21.88908	53.01217	0	0	73.64015	0	53.80054	0	0	17.90926	41.78905	29.99069
	Std	0	34.17988	6.678404	0	0	54.01573	0	26.56134	2.53E−14	0	6.328985	15.30696	19.0714
	Median	0	34.82353	59.66433	0	0	179.0786	0	98.10387	0	0	26.86388	59.20266	56.10198
	ET	2.538358	6.205782	5.169585	0.716117	3.369601	1.28455	0.76484	3.217982	1.464147	2.281739	3.880075	0.667393	0.898574
	Rank	1	4	7	1	1	9	1	8	2	1	3	6	5
${F}_{10}$	Mean	8.88E−16	7.25E−07	4.66E−07	3.983745	4.09E−15	1.701773	3.73E−15	0.501389	1.62E−14	4.26E−15	7.76E−09	3.003923	3.519331
	Best	8.88E−16	4.64E−07	2.39E−07	8.88E−16	8.88E−16	1.51E−14	8.88E−16	0.082022	1.51E−14	8.88E−16	5.52E−09	1.340457	2.591243
	Std	0	2.12E−07	1.26E−07	8.174475	1.09E−15	1.597276	2.47E−15	0.532278	2.33E−15	7.94E−16	1.64E−09	1.002929	0.345136
	Median	8.88E−16	6.94E−07	4.57E−07	8.88E−16	4.44E−15	2.542805	4.44E−15	0.150768	1.51E−14	4.44E−15	7.64E−09	2.997188	3.461528
	ET	2.493472	6.140622	6.096217	0.74041	3.261045	1.263234	0.810686	3.316977	1.495081	2.358528	3.971348	0.670133	0.930872
	Rank	1	8	7	13	3	10	2	9	5	4	6	11	12
${F}_{11}$	Mean	0	0.001108	2.13E−10	0	0	0.005297	0.003099	0.393366	0.002687	0	8.937934	0.155581	1.52779
	Best	0	5.91E−11	3.33E−12	0	0	0	0	0.201826	0	0	4.884369	0.012736	1.217033
	Std	0	0.003615	6.53E−10	0	0	0.007265	0.013861	0.087275	0.006679	0	3.012101	0.147889	0.242677
	Median	0	1.8E−10	3.01E−11	0	0	0	0	0.398108	0	0	8.251011	0.116847	1.468467
	ET	3.256439	6.444046	7.498936	1.002643	3.606022	1.436769	1.091555	3.741729	1.735395	3.150524	4.587756	0.913732	1.177281
	Rank	1	3	2	1	1	6	5	8	4	1	10	7	9
${F}_{12}$	Mean	1.96E−09	2.18E−12	3.95E−13	8.45E−09	1.99E−10	6.305474	0.005405	1.231756	0.035924	0.079774	0.28432	1.662913	0.201968
	Best	3.96E−10	6.95E−13	1.16E−13	3.34E−10	5.48E−10	0.264734	0.001423	0.000924	0.013184	0.056662	6.02E−19	0.000169	0.058181
	Std	1.23E−09	8.82E−13	2.71E−13	1.47E−08	7.9E−09	3.766997	0.004457	1.21416	0.013328	0.020464	0.363627	1.723156	0.125589
	Median	1.73E−09	2.14E−12	3.19E−13	4.05E−09	1.98E−09	6.493901	0.00338	0.8514	0.037334	0.075443	0.103669	0.960977	0.175433
	ET	9.718521	7.946868	8.937058	3.303009	7.479572	3.532742	3.727992	7.699429	4.874407	9.291213	6.513698	2.878026	3.056695
	Rank	3	2	1	5	4	13	6	11	7	8	10	12	9
${F}_{13}$	Mean	5.05E−08	5.11E−11	2.27E−12	0.114684	0.002561	2.648762	0.246551	0.02783	0.487249	1.052281	0.006549	4.893821	2.342733
	Best	6.18E−09	1.42E−11	5.44E−13	1.57E−08	1.42E−09	1.949438	0.031826	0.009919	0.100058	0.500205	5.54E−18	0.012249	1.205092
	Std	7.54E−08	2.68E−11	1.16E−12	0.134062	0.004919	0.381937	0.207692	0.012327	0.220038	0.253877	0.010866	4.946219	0.868383
	Median	2.26E−08	4.68E−11	2.35E−12	0.097372	3.66E−09	2.494033	0.221514	0.027171	0.583629	1.087803	1.83E−17	4.264547	2.307728
	ET	9.253492	7.923011	8.270599	3.27416	7.552763	3.531932	3.678267	7.733775	4.754169	8.792697	6.567627	2.870053	3.094304
	Rank	3	2	1	7	4	12	8	6	9	10	5	13	11
Sum rank		12	20	20	34	17	60	27	50	38	36	47	58	52
Mean rank		2	3.333333	3.333333	5.666667	2.833333	10	4.5	8.333333	6.333333	6	7.833333	9.666667	8.666667
Total rank		1	3	3	5	2	12	4	9	7	6	8	11	10

### Evaluation fixed-dimensional multimodal objective function

The results of the CBOA and competitor algorithms for the fixed-dimensional multimodal functions ${F}_{14}$ to ${F}_{23}$ are presented in Table 7. The optimization results show that the CBOA, based on the “mean index’’, alone is the best optimizer to tackle the functions ${F}_{14}$, ${F}_{20}$, and ${F}_{18}$.

**Table 7:** Results of optimization of the CBOA and competitor metaheuristics on fixed-dimensional multimodal function.

		CBOA	CMA	DE	HBA	MPA	TSA	WOA	MVO	GWO	TLBO	GSA	PSO	GA
${F}_{14}$	Mean	0.998004	6.468644	1.097407	1.974522	0.998004	9.754139	1.692637	0.998004	5.011135	1.29562	3.977845	3.596373	1.001145
	Best	0.998004	1.149956	0.998004	0.998004	0.998004	0.998004	0.998004	0.998004	0.998004	0.998004	0.998004	0.998004	0.998004
	Std	6.62E−17	3.807911	0.305955	3.005658	7.2E−17	5.084877	0.916108	5.7E−12	4.396499	0.72687	2.839185	3.748318	0.010488
	Median	0.998004	6.574582	0.998004	0.998004	0.998004	12.67051	0.998004	0.998004	2.982105	0.998004	2.890313	0.998004	0.998004
	ET	8.263481	7.439983	11.49533	5.927204	12.84467	5.237797	6.893766	9.8159	5.559464	15.56081	6.505227	4.960642	5.409746
	Rank	1	12	5	8	2	13	7	3	11	6	10	9	4
${F}_{15}$	Mean	0.000344	0.0034	0.000686	0.005788	0.006988	0.006334	0.000594	0.002723	0.002313	0.002482	0.002868	0.002183	0.010056
	Best	0.000308	0.001084	0.000451	0.000307	0.00032	0.000308	0.000309	0.000308	0.000307	0.000309	0.001183	0.000307	0.001759
	Std	6.05E−16	3.94E−14	1.43E−15	9.18E−14	1.03E−13	1.39E−13	3.77E−15	6.04E−14	6.17E−14	6.12E−14	2.03E−14	4.98E−14	9.09E−14
	Median	0.00032	0.002026	0.000678	0.000765	0.000772	0.000487	0.000459	0.000724	0.000307	0.000316	0.002326	0.000307	0.005585
	ET	1.021564	4.023251	5.574032	0.536539	1.728231	0.564211	0.637955	1.323245	0.60876	1.95416	1.90813	0.435123	0.717282
	Rank	1	9	3	10	12	11	2	7	5	6	8	4	13
${F}_{16}$	Mean	− 1.03163	− 1.03163	− 1.03163	− 1.03163	− 1.03163	− 1.02847	− 1.03163	− 1.03163	− 1.03163	− 1.03163	− 1.03163	− 1.03163	− 1.03163
	Best	− 1.03163	− 1.03163	− 1.03163	− 1.03163	− 1.03163	− 1.03163	− 1.03163	− 1.03163	− 1.03163	− 1.03163	− 1.03163	− 1.03163	− 1.03163
	Std	1.41E−16	2.28E−15	2.28E−15	2.04E−15	1.84E−15	9.74E−14	8.92E−12	3.45E−11	4.44E−14	1.32E−10	1.44E−10	1.14E−10	1.62E−10
	Median	− 1.03163	− 1.03163	− 1.03163	− 1.03163	− 1.03163	− 1.03163	− 1.03163	− 1.03163	− 1.03163	− 1.03163	− 1.03163	− 1.03163	− 1.03163
	ET	1.341562	3.836853	5.448002	0.452356	1.663918	0.497087	0.576034	1.14112	0.489379	1.733055	1.756584	0.328678	0.632265
	Rank	1	1	1	1	1	7	2	4	3	6	1	1	5
${F}_{17}$	Mean	0.397887	0.397887	0.397887	0.397887	0.397887	0.397904	0.397888	0.397887	0.397888	0.400341	0.397887	0.672818	0.409121
	Best	0.397887	0.397887	0.397887	0.397887	0.397887	0.397888	0.397887	0.397887	0.397887	0.397899	0.397887	0.397887	0.397887
	Std	0	0	0	0	0	1.88E−16	1.68E−17	7.31E−19	4.57E−18	1.03E−13	0	6.27E−12	4.94E−13
	Median	0.397887	0.397887	0.397887	0.397887	0.397887	0.397897	0.397888	0.397887	0.397888	0.397956	0.397887	0.397887	0.397891
	ET	2.015974	3.879041	5.777031	0.42436	1.65395	0.482376	0.567438	1.066481	0.479291	1.579686	2.054971	0.281283	0.577906
	Rank	1	1	1	1	1	5	4	2	3	6	1	8	7
${F}_{18}$	Mean	3	3	3	3	3	8.400016	3.000012	3	3.000008	3.000002	3	3	3.001894
	Best	3	3	3	3	3	3	3	3	3	3	3	3	3
	Std	1.19E−17	7.96E−27	4.2E−27	6.98E−27	1.07E−26	1.88E−10	1.72E−16	6.04E−18	6.08E−17	2.99E−17	1.92E−26	2.82E−26	3.37E−14
	Median	3	3	3	3	3	3.000008	3.000006	3	3.000006	3	3	3	3.00035
	ET	2.652104	3.793405	5.677689	0.379509	1.501908	0.467816	0.494053	1.017728	0.412652	1.523375	1.922881	0.264965	0.567245
	Rank	1	1	1	1	1	9	7	4	6	5	3	2	8
${F}_{19}$	Mean	− 3.86278	− 3.86278	− 3.86278	− 3.86121	− 3.86278	− 3.86273	− 3.86002	− 3.86278	− 3.86112	− 3.86175	− 3.86278	− 3.86278	− 3.86272
	Best	− 3.86278	− 3.86278	− 3.86278	− 3.86278	− 3.86278	− 3.86278	− 3.86277	− 3.86278	− 3.86278	− 3.86269	− 3.86278	− 3.86278	− 3.86278
	Std	1.86E−16	2.28E−26	2.28E−26	3.23E−14	2.28E−26	3.95E−16	2.86E−14	9.56E−19	3.07E−14	2.17E−14	1.95E−26	2.03E−26	1.23E−15
	Median	− 3.86278	− 3.86278	− 3.86278	− 3.86278	− 3.86278	− 3.86274	− 3.86119	− 3.86278	− 3.86275	− 3.86252	− 3.86278	− 3.86278	− 3.86277
	ET	3.1452930	4.084194	5.110906	0.562827	1.844683	0.61375	0.691191	1.336033	0.618499	2.048364	2.215913	0.434463	0.743604
	Rank	1	1	1	6	1	3	8	2	7	5	1	1	4
${F}_{20}$	Mean	− 3.322	− 3.26255	− 3.3099	− 3.24296	− 3.322	− 3.25141	− 3.24583	− 3.23852	− 3.26038	− 3.27726	− 3.322	− 3.28245	− 3.19973
	Best	− 3.322	− 3.322	− 3.322	− 3.322	− 3.322	− 3.32129	− 3.32194	− 3.322	− 3.32199	− 3.31657	− 3.322	− 3.322	− 3.30608
	Std	1.78E−16	6.1E−13	3.65E−13	7.89E−13	4.08E−27	1.16E−12	1.26E−12	5.61E−13	8.32E−13	4.92E−13	4.2E−27	7.22E−13	7.02E−13
	Median	− 3.32199	− 3.26255	− 3.322	− 3.2031	− 3.322	− 3.31986	− 3.32047	− 3.2029	− 3.32199	− 3.30137	− 3.322	− 3.322	− 3.19502
	ET	2.546312	4.168919	5.611372	0.591109	1.961109	0.666685	0.717402	1.586025	0.719107	2.123327	2.376263	0.464815	0.75444
	Rank	1	5	2	9	1	7	8	10	6	4	1	3	11
${F}_{21}$	Mean	− 10.1532	− 6.79155	− 9.88592	− 9.31345	− 10.1532	− 6.3241	− 8.49107	− 7.87273	− 9.13554	− 6.28747	− 6.70335	− 5.2634	− 5.65214
	Best	− 10.1532	− 10.1532	− 10.1532	− 10.1532	− 10.1532	− 10.1049	− 10.1531	− 10.1532	− 10.1531	− 9.91428	− 10.1532	− 10.1532	− 9.91577
	Std	1.52E−16	3.81E−11	1.13E−11	2.6E−11	2.19E−15	3.21E−11	2.65E−11	2.59E−11	2.09E−11	1.83E−11	3.6E−11	3.08E−11	2.71E−11
	Median	− − 10.1532	− 10.1532	− 10.1532	− 10.1532	− 10.1532	− 4.86615	− 10.1479	− 10.1531	− 10.1528	− 6.73438	− 7.90835	− 5.0552	− 5.63103
	ET	2.146528	4.163368	5.270078	0.74175	2.364909	0.742612	0.896988	1.823964	0.775067	2.447205	2.386266	0.586512	0.87221
	Rank	1	8	3	4	2	10	6	7	5	11	9	13	12
${F}_{22}$	Mean	− 10.4029	− 10.0211	− 10.4006	− 9.25738	− 10.4029	− 7.26663	− 9.11739	− 9.60765	− 10.1367	− 8.07294	− 10.1831	− 7.0107	− 6.10828
	Best	− 10.4029	− 10.4029	− 10.4029	− 10.4029	− 10.4029	− 10.3162	− 10.4029	− 10.4029	− 10.4029	− 9.75254	− 10.4029	− 10.4029	− 10.017
	Std	1.12E−16	1.71E−11	9.58E−14	2.8E−11	3.65E−15	3.38E−11	2.58E−11	1.94E−11	1.19E−11	1.59E−11	9.83E−12	3.85E−11	2.61E−11
	Median	− 10.4029	− 10.4029	− 10.4029	− 10.4029	− 10.4029	− 10.0266	− 10.3988	− 10.4029	− 10.4025	− 8.49275	− 10.4029	− 10.4029	− 6.26211
	ET	2.165974	4.267036	5.149368	0.828738	2.47023	0.830113	0.991285	2.111943	0.881028	2.686496	2.167587	0.666191	0.974745
	rank	1	6	3	8	2	11	9	7	5	10	4	12	13
${F}_{23}$	Mean	− 10.5364	− 9.72522	− 10.4914	− 9.79563	− 10.5364	− 6.27469	− 8.13732	− 9.05875	− 9.58937	− 7.44495	− 10.3652	− 6.10808	− 6.58071
	Best	− 10.5364	− 10.5364	− 10.5364	− 10.5364	− 10.5364	− 10.4786	− − 10.5357	− 10.5364	− 10.5363	− 9.81289	− 10.5364	− 10.5364	− 9.95588
	Std	1.63E−16	2.5E−11	2.01E−12	2.29E−11	2.7E−16	3.9E−11	3.08E−11	2.69E−11	2.37E−11	2.11E−11	7.66E−12	3.77E−11	2.75E−11
	Median	− 10.5364	− 10.5364	− 10.5364	− 10.5364	− 10.5364	− 5.12692	− 10.5292	− 10.5363	− 10.5357	− 8.67305	− 10.5364	− 3.83543	− 7.68126
	ET	2.314952	4.380511	5.227086	0.966058	2.820167	0.956041	1.130458	2.209019	1.011938	3.055197	2.329461	0.801659	1.083483
	Rank	1	6	3	5	2	12	9	8	7	10	4	13	11
Sum rank		10	50	23	53	25	88	62	54	58	69	42	66	88
Mean rank		1	5	2.3	5.3	2.5	8.8	6.2	5.4	5.8	6.9	4.2	6.6	8.8
Total rank		1	5	2	6	3	12	9	7	8	11	4	10	12

In the other cases where the CBOA has the same conditions in terms of the “mean index’’, it performs more efficiently than the alternative algorithms due to better values for the “std index’’. Analysis of the simulation results shows that the CBOA performs better than competitor algorithms and has a remarkable ability to strike a balance between exploration and exploitation.

In other cases where the CBOA has the same conditions in terms of the “mean index’’, it has more efficient performance than the alternative algorithms due to better values for the “std index’’. Analysis of the simulation results shows that the CBOA performs better than competitor algorithms and has a remarkable ability to strike a balance between exploration and exploitation.

**Figure 2:**
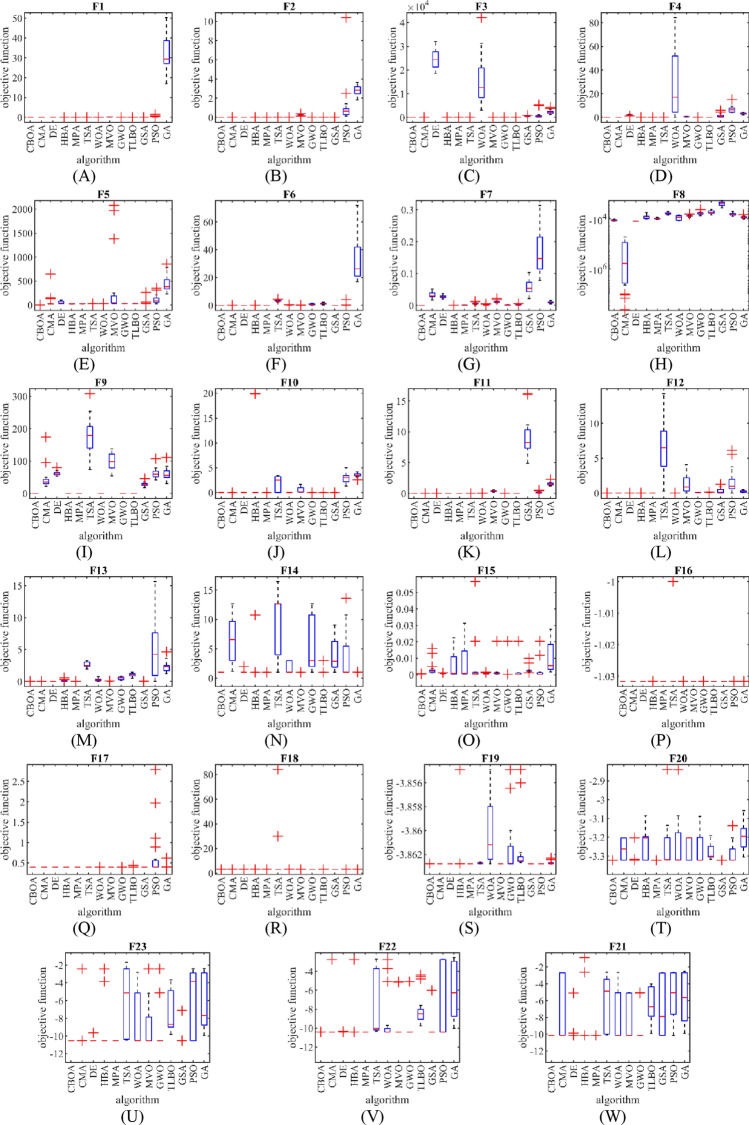
The boxplot diagram of CBOA and competitor algorithms performances on ${F}_{1}$ to ${F}_{23}$.

The performance of CBOA and competitor algorithms in evaluating the benchmark functions ${F}_{1}$ to ${F}_{23}$ is shown in Fig. 2 using the box plot diagrams.

### Statistical analysis

In this subsection, a statistical analysis of the performance of the CBOA compared to competitor algorithms is provided to determine whether the superiority of the CBOA is statistically significant. To provide this analysis, the Wilcoxon test of rank sums^32^ with the significance level $5\%$ has been used. In this test, the values of the “$p$-value’’ indicate whether there is a significant difference between the means of the two data samples (thus, if the “$p$-value’’ is less than 0.05, then between two data samples is significant difference). The results of the Wilcoxon test of rank sums for the CBOA and competitor algorithms are released in Table 8. Consequently, since all values obtained for the $p$-value are less than 0.05, the CBOA has a significant statistical superiority over all twelve compared algorithms.

**Table 8:** Results of Wilcoxon test of rank sums.

Compared algorithm	Objective function type		
	Unimodal	High-dimensional multimodal	Fixed-dimensional multimodal
CBOA vs. CMA	1.01E−24	7.53E−03	5.75E−03
CBOA vs. DE	1.01E−24	8.25E−03	2.75E−07
CBOA vs. HBA	1.21E−11	3.91E−11	4.14E−06
CBOA vs. MPA	1.01E−24	0.170913	1.63E−14
CBOA vs. TSA	1.01E−24	1.28E−19	1.88E−32
CBOA vs. WOA	2.49E−24	5.46E−10	2.36E−31
CBOA vs. MVO	1.01E−24	1.97E−21	9.13E−25
CBOA vs. GWO	1.01E−24	3.55E−16	5.16E−25
CBOA vs. TLBO	1.01E−24	1.04E−14	1.96E−30
CBOA vs. GSA	1.01E−24	3.61E−17	0.0169847
CBOA vs. PSO	1.01E−24	1.97E−21	0.0201922
CBOA vs. GA	1.01E−24	1.97E−21	1.2E−33

### Sensitivity analysis

The proposed CBOA is a stochastic optimizer that can achieve the optimal solution by using its members’ search power in an iteration-based process. Therefore, the values of the parameters $N$ and $T$, which represent the number of CBOA members and the total number of iterations of the algorithm, respectively, affect the performance of the CBOA. To study this effect, we analyze the sensitivity of CBOA to changes in values of the $N$ and $T$ parameters in this subsection.

**Figure 3:**
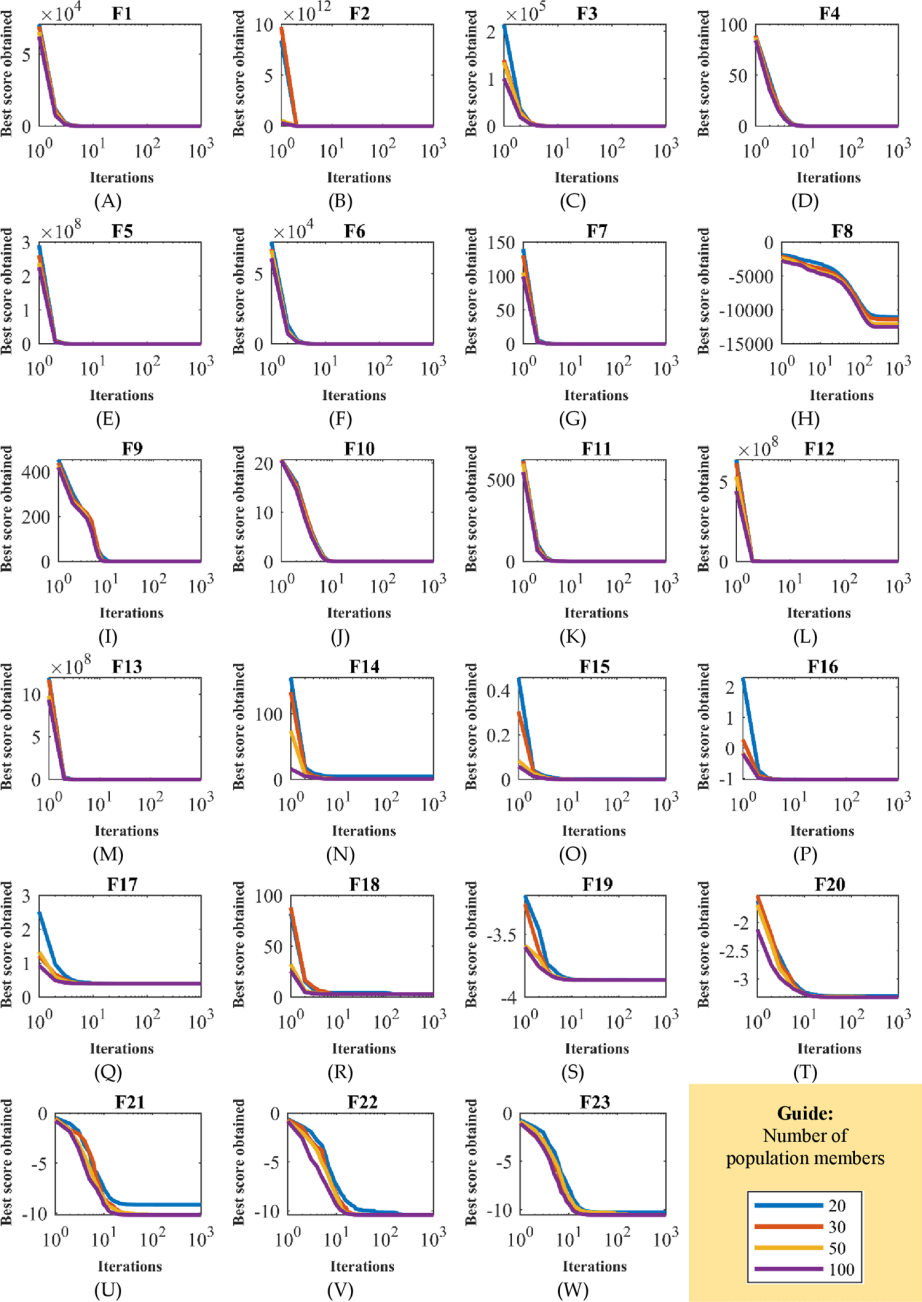
CBOA convergence curves in the study of sensitivity analysis to parameter $N$.

In the first study, to analyze the sensitivity of CBOA to the parameter $N$, the proposed algorithm in independent performance for different values of the parameter $N$ equal to 20, 30, 50, and 100 is used to optimize the functions of ${F}_{1}$ to ${F}_{23}$. Results of this analysis are presented in Table 9, and CBOA convergence curves to optimize these objective functions under the influence of the changes of the parameter $N$ are shown in Fig. 3. Based on simulation results obtained from the sensitivity analysis of the parameter $N$, it is clear that CBOA presents similar results in most objective functions when the parameter $N$ is changed, indicating that the CBOA is less affected by the parameter $N$. In other cases of the objective functions, it can be seen that when the value of the parameter $N$ increases, then the values of objective functions decrease.

**Table 9:** Results of CBOA sensitivity analysis to parameter $N$.

Objective functions	Number of population members			
	20	30	50	100
${F}_{1}$	0	0	0	0
${F}_{2}$	0	0	0	0
${F}_{3}$	0	0	0	0
${F}_{4}$	0	0	0	0
${F}_{5}$	0.000378	0.000306	0.000144	4.23E−05
${F}_{6}$	0	0	0	0
${F}_{7}$	7.06E−05	4.26E−05	2.97E−05	1.60E−05
${F}_{8}$	− 11,071	− 11,416.7	− 12,119.4	− 12,504.3
${F}_{9}$	0	0	0	0
${F}_{10}$	2.84E−15	8.88E−16	8.88E−16	8.88E−16
${F}_{11}$	0	0	0	0
${F}_{12}$	5.62E−09	1.96E−09	4.14E−10	1.16E−10
${F}_{13}$	7.12E−08	5.05E−08	6.46E−09	1.49E−09
${F}_{14}$	4.194612	0.998004	0.998004	0.998004
${F}_{15}$	0.001348	0.000344	0.000332	0.000321
${F}_{16}$	− 1.03163	− 1.03163	− 1.031630	− 1.031630
${F}_{17}$	0.397892	0.397887	0.397887	0.397887
${F}_{18}$	3	3	3	3
${F}_{19}$	− 3.86235	− 3.86278	− 3.86278	− 3.8628
${F}_{20}$	− 3.30339	− 3.3220	− 3.3220	− 3.3220
${F}_{21}$	− 9.13349	− 10.1532	− 10.1532	− 10.1532
${F}_{22}$	− 10.4027	− 10.4029	− 10.4029	− 10.4029
${F}_{23}$	− 10.2659	− 10.5364	− 10.5364	− 10.5364

**Figure 4:**
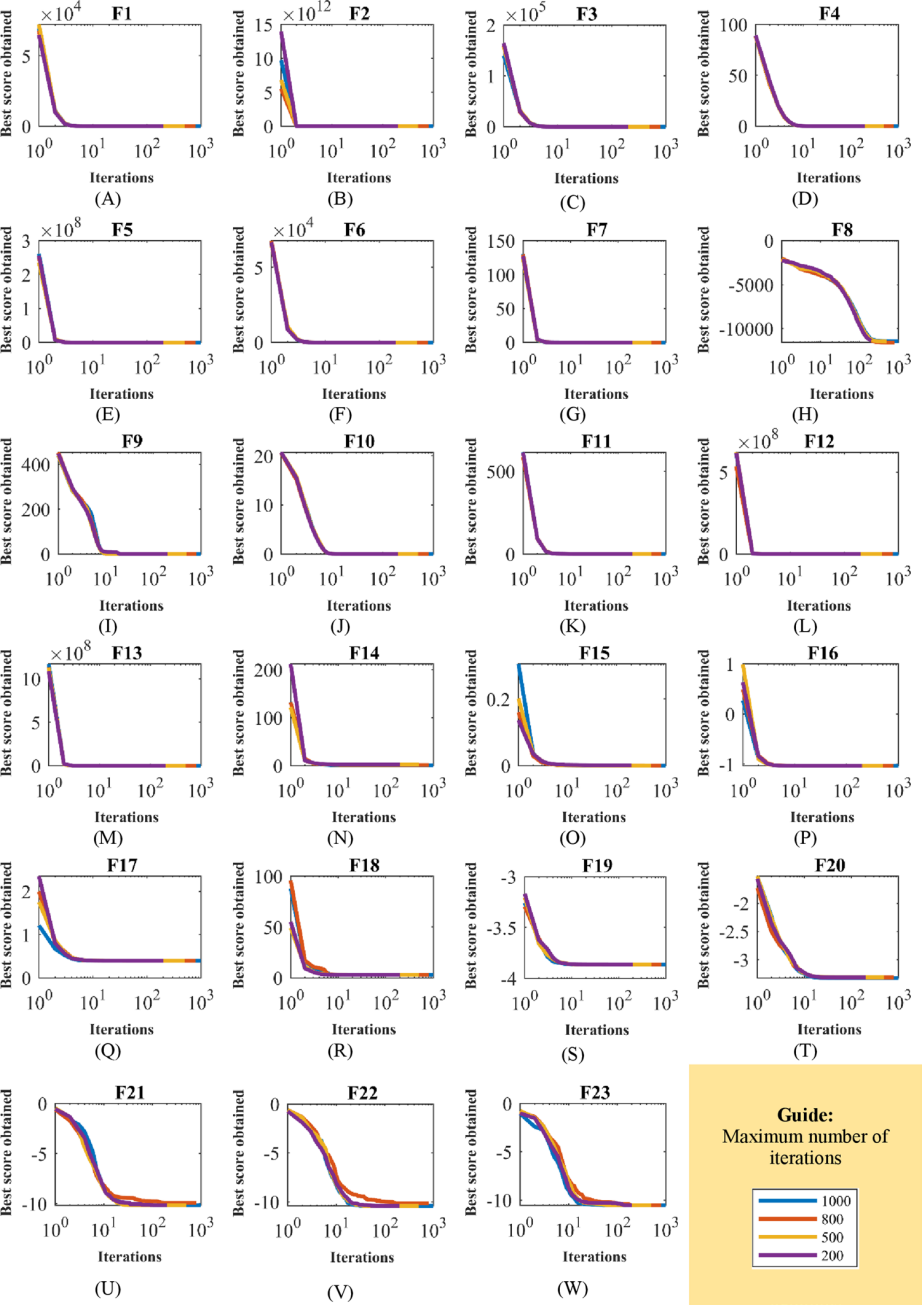
CBOA convergence curves in the study of sensitivity analysis to parameter $T$.

In the second study, to analyze the sensitivity of CBOA to the parameter $T$, the proposed method is implemented in independent performances for different values of the parameter $T$ equal to 200, 500, 800, and 1000 on the objective functions ${F}_{1}$ to ${F}_{23}$. The results of this analysis are reported in Table 10, and the CBOA convergence curves affected by this study are plotted in Fig. 4. What is clear from the results of the CBOA sensitivity analysis to changes in the parameter $T$ is that by increasing the values of the parameter $T$, the performance of CBOA is improved and as a result, the values of objective functions are decreased.

**Table 10:** Results of the CBOA sensitivity analysis to parameter $T$.

Objective functions	Maximum number of iterations			
	200	500	800	1000
${F}_{1}$	1.30E−146	0	0	0
${F}_{2}$	5.04E−76	8.40E−192	0	0
${F}_{3}$	1.10E−127	0	0	0
${F}_{4}$	2.46E−72	6.60E−184	4.20E−294	0
${F}_{5}$	0.081809	0.003117	0.000586	0.000306
${F}_{6}$	0	0	0	0
${F}_{7}$	0.00019	7.75E−05	5.91E−05	4.26E−05
${F}_{8}$	− 11,338.3	− 11,357.2	− 11,406.8	− 11,416.7
${F}_{9}$	0	0	0	0
${F}_{10}$	2.31E−15	1.42E−15	1.15E−15	8.88E−16
${F}_{11}$	0	0	0	0
${F}_{12}$	3.84E−05	3.66E−08	3.81E−09	1.96E−09
${F}_{13}$	0.000369	3.68E−07	6.48E−08	5.05E−08
${F}_{14}$	2.480011	2.130218	1.542754	0.998004
${F}_{15}$	0.001379	0.000377	0.000376	0.000344
${F}_{16}$	− 1.03163	− 1.03163	− 1.03163	− 1.03163
${F}_{17}$	0.39789	0.397888	0.397889	0.397887
${F}_{18}$	3	3	3	3
${F}_{19}$	− 3.86235	− 3.86243	− 3.86267	− 3.86278
${F}_{20}$	− 3.31573	− 3.31604	− 3.31603	− 3.3220
${F}_{21}$	− 10.1531	− 10.1531	− 10.1532	− 10.1532
${F}_{22}$	− 10.4027	− 10.4028	− 10.4029	− 10.4029
${F}_{23}$	− 10.5361	− 10.5363	− 10.5364	− 10.5364

## Discussion

Metaheuristic algorithms are random approaches with which their main idea employed in the optimization process is random search in the problem-solving space. This random search at both local and global levels is the key to the success of metaheuristic algorithms. In optimization studies, the local search power, which indicates the potential for exploitation, causes the algorithm to look for better solutions around promising candidate solutions and move closer to the optimal global solution. The capability of the “exploitation phase’’ in metaheuristic algorithms is best tested when implemented on functions that have only one main solution. Unimodal functions with this feature are good options for evaluating the exploitation ability. The optimization results of unimodal functions ${F}_{1}$ to ${F}_{7}$ indicate the high exploitation capability of CBOA, especially in handling ${F}_{1}$ to ${F}_{4}$, and ${F}_{6}$. Therefore, the simulation finding from the results of the unimodal functions is that the CBOA has high efficiency in local search and a high potential for exploitation.

The power of global search, which demonstrates the exploration potential of metaheuristic algorithms, allows the algorithm to scan different areas of the search space to discover the optimal global area. The capability of the “exploration phase’’ in metaheuristic algorithms designed for optimization can best be evaluated using optimization problems with several local optimal solutions. Therefore, high-dimensional multimodal functions are a good choice for evaluating exploration ability. The implementation results of CBOA and competitor algorithms on functions ${F}_{8}$ to ${F}_{13}$ show the high exploration ability of CBOA to global search in various areas of the problem-solving space. This CBOA capability is especially evident in the optimization results of the functions ${F}_{9}$ and ${F}_{11}$. The finding from simulations of the CBOA and competitor algorithms on the fixed-dimensional multimodal functions ${F}_{8}$ to ${F}_{13}$ is that the CBOA, with its high power in global search and exploration, can avoid getting stuck in locally optimal solutions and identify the main optimal region.

The critical point in the capability of metaheuristic algorithms is that in addition to having the desired ability in exploitation and exploration, there must be a balance between these two capabilities so that the algorithm can find the main optimal region and converge towards the global optimal. Fixed-dimensional multimodal functions are good options for testing the ability of metaheuristic algorithms to strike a balance between exploitation and exploration. Optimizing the ${F}_{14}$ to ${F}_{23}$ functions shows that the CBOA has a high potential to strike a balance between exploitation and exploration. Based on the fixed-dimension multimodal function optimization results, CBOA, with its ability to balance exploitation and exploration, can first discover the main optimal region by global search without getting entangled in locally optimal solutions, then converge to the global optimum by local search. The execution time of CBOA and competing algorithms in optimizing each objective function shows that CBOA is faster than some competing algorithms. But some other competing algorithms, although faster, did not converge to the desired results. Therefore, CBOA has an acceptable execution time when optimizing the objective functions.

The simulation findings show that CBOA has a high quality in exploitation, exploration, and balance between them, which has led to its superior performance compared to similar competing algorithms.

### Evaluation CEC 2017 test suite

To analyze the capability of the proposed CBOA approach in complex optimization problems, the proposed algorithm is implemented on the CEC 2017 test suite. This set includes three unimodal objective functions ${C}_{1}$ to ${C}_{3}$, seven multimodal objective functions ${C}_{4}$ to ${C}_{10}$, ten hybrid objective functions ${C}_{11}$ to ${C}_{20}$, and ten composition objective functions ${C}_{21}$ to ${C}_{30}$. Complete information and details of the CEC 2017 test suite are described in Ref.^33^. The ${C}_{2}$ function has been removed from the CEC 2017 set due to its unstable behavior. The implementation results of CBOA and competitor algorithms on the CEC 2017 test suite are published in Table 11. Based on the analysis of simulation results, it is clear that the proposed CBOA approach is the first best optimizer for ${C}_{1}$, ${C}_{3}$, ${C}_{4}$, ${C}_{6}$ to ${C}_{8}$, ${C}_{10}$ to ${C}_{20}$, ${C}_{22}$, ${C}_{24}$, ${C}_{25}$, ${C}_{27}$, and ${C}_{28}$ functions compared to competitor algorithms.

**Table 11:** Assessment results of the IEEE CEC 2017 objective functions.

		CBOA	CMA	DE	HBA	MPA	TSA	WOA	MVO	GWO	TLBO	GSA	PSO	GA
*C*_1_	Mean	101.2	3108.912	3.25E+09	1.1E+10	110.9548	1.88E+09	6,961,876	8111.161	95,212,633	1.59E+08	799.093	3387.4	12,792,740
	Std	1.35E−05	3271.819	7.25E+08	1.63E+09	4.824002	1.64E+09	1,733,856	3184.727	1.68E+08	1.51E+08	789.2879	4480.944	4,907,280
	ET	2.669477	1.060814	2.741406	6.984978	3.742614	1.420908	1.267498	2.243143	1.496543	5.170083	3.504213	1.654582	2.221838
	Rank	1	4	12	13	2	11	7	6	9	10	3	5	8
${C}_{3}$	Mean	101.2	247,961.2	3.87E+09	1.06E+10	112.0599	4.74E+09	5,042,647	6350.138	92,201,634	85,680,111	606.3214	1660.034	13,171,473
	Std	9.42E-06	456,774.1	1.01E+09	2.25E+09	2.827652	3.07E+09	2,196,191	2081.009	1.82E+08	30,546,657	817.5146	1234.834	12,142,481
	ET	2.513491	1.052585	2.633707	6.744735	3.241847	1.349695	1.157729	1.744399	1.384935	5.078677	3.00369	1.505888	1.589703
	Rank	1	6	11	13	2	12	7	5	10	9	3	4	8
${C}_{4}$	Mean	303.6	470.0877	6557.026	10,391.17	303.6	12,068.64	1846.57	303.6589	3291.494	763.5923	11,049.4	334.5206	15,922.03
	Std	4.64E-14	257.8634	1675.101	3802.403	4.61E-11	5302.915	1378.411	0.052941	2170.273	199.4561	3332.523	7.473065	10,711.33
	ET	2.607193	1.044459	2.636024	6.724772	3.308203	1.369852	1.187209	1.660072	1.4016	5.100191	2.948115	1.373677	1.653028
	Rank	1	5	9	10	2	12	7	3	8	6	11	4	13
${C}_{5}$	Mean	414.3606	412.1832	714.3499	1431.825	404.8017	595.334	431.9613	408.4013	417.4771	414.7045	409.7174	426.738	420.6968
	Std	1.926314	0.618212	105.8268	461.7888	0.003466	113.116	34.96135	1.853898	11.96904	0.592914	1.245504	36.41708	3.195525
	ET	2.622783	1.029484	2.639928	6.76956	3.440196	1.349224	1.182745	1.778327	1.407783	5.035687	2.897966	1.447792	1.605425
	Rank	5	4	12	13	1	11	10	2	7	6	3	9	8
${C}_{6}$	Mean	513.8433	524.1366	551.5381	585.3227	558.7895	576.1053	550.596	531.7634	536.8321	543.0554	564.6515	536.3466	536.4685
	Std	1.321151	2.951072	7.033816	17.97259	32.28185	25.69122	27.24099	12.61261	30.47843	4.324143	8.676174	20.46161	5.170309
	ET	2.752942	1.049288	2.710091	6.841018	3.405888	1.443739	1.256369	1.835352	1.504136	5.302203	3.010028	1.466968	1.725688
	Rank	1	2	9	13	10	12	8	3	6	7	11	4	5
${C}_{7}$	Mean	607.2005	608.0567	625.7126	651.7765	667.2729	634.3909	632.5699	609.5542	608.4342	614.7151	626.0413	615.3363	618.4357
	Std	0.000362	0.92784	2.462733	3.674113	23.00177	11.97368	17.37205	1.890297	0.509057	2.688245	16.833	8.893649	3.689074
	ET	3.280764	1.266268	2.926952	7.050865	3.8106	1.644312	1.449149	2.069021	1.741808	5.997119	3.147501	1.675329	1.895828
	Rank	1	2	8	12	13	11	10	4	3	5	9	6	7
${C}_{8}$	Mean	724.5491	747.7368	784.9913	821.7705	751.0584	848.1839	775.4688	741.2936	735.9656	764.4844	750.7485	743.3355	747.8592
	Std	1.089227	23.78253	9.986853	13.32549	41.61996	38.82131	21.52091	15.21458	13.1403	6.219753	27.77594	9.382105	7.716694
	ET	2.998274	1.138406	2.809196	7.021971	3.545909	1.495996	1.323537	1.910198	1.575269	5.486644	3.073433	1.453913	1.751502
	Rank	1	5	11	12	8	13	10	3	2	9	7	4	6
${C}_{9}$	Mean	832.4164	818.4122	844.1283	868.3161	820.6759	862.389	849.3245	822.4432	826.8465	850.8005	831.2483	834.4307	827.8797
	Std	5.688899	5.288314	6.336957	8.355853	3.767465	17.32405	14.07283	4.143453	4.726786	8.355722	7.284064	7.299423	5.822149
	ET	2.763709	1.115557	2.733269	6.851644	3.439406	1.436942	1.277088	1.851924	1.524415	5.569266	2.968746	1.437815	1.79004
	Rank	7	1	9	13	2	12	10	3	4	11	6	8	5
${C}_{10}$	Mean	910.8	940.4649	1208.774	1532.356	910.8	1437.488	1431.553	911.6781	923.8767	923.7591	982.0398	915.4483	916.4008
	Std	0	35.31109	75.87706	108.8322	2.37E-08	237.4952	268.5616	1.690314	16.73681	6.150759	25.37577	5.975467	3.11235
	ET	3.05801	1.160071	2.777339	6.882568	3.556943	1.515257	1.347831	2.134891	1.548243	5.434826	3.089326	1.443124	1.820621
	Rank	1	8	10	13	2	12	11	3	7	6	9	4	5
${C}_{11}$	Mean	1286.22	1626.486	2049.993	2724.324	1453.429	2131.957	2123.707	1858.522	1798.419	2283.273	2398.428	2037.753	1787.846
	Std	114.1865	402.2849	117.5223	268.4464	61.60307	302.0841	577.3136	434.7159	209.3108	313.6769	203.0563	353.1798	324.2988
	ET	2.94732	1.080666	2.743516	6.831648	3.497469	1.500188	1.270246	2.487187	1.559109	5.573976	3.039886	1.479923	1.764071
	Rank	1	3	8	13	2	10	9	6	5	11	12	7	4
${C}_{12}$	Mean	1115.37	1137.514	3098.063	4239.627	1249.513	5838.905	1168.437	1143.017	1173.114	1168.387	1155.684	1160.384	2503.703
	Std	0.588853	6.537338	592.0687	2445.697	38.35352	110.5968	30.10533	23.48768	53.94393	16.13176	22.65592	15.99788	2599.637
	ET	2.732119	1.070258	2.737073	6.951227	3.429757	1.440446	1.239599	1.850855	1.484072	5.379859	2.98204	1.487207	1.780409
	Rank	1	2	11	12	9	13	7	3	8	6	4	5	10
${C}_{13}$	Mean	1242.671	2371.454	1.92E+08	7.67E+08	1376.876	1,129,898	2,558,443	1,118,413	1,538,184	5,491,479	1,108,928	8691.271	657,472.1
	Std	34.81118	1339.775	1.48E+08	5.92E+08	22.58375	377,867.2	1,886,333	1,618,529	1,039,514	4,371,092	575,601.5	5641.408	398,429.3
	ET	2.750974	1.159016	2.719459	6.770772	3.412868	1.434666	1.227595	1.963578	1.51061	5.446068	2.996924	1.515922	1.768249
	Rank	1	3	12	13	2	8	10	7	9	11	6	4	5
${C}_{14}$	Mean	1321.672	1345.379	9,353,580	37,362,843	1452.289	13,745.31	8138.937	7214.729	11,095.13	18,079.89	10,847.64	7098.111	59,078.95
	Std	0.905728	23.47823	14,486,856	57,908,805	53.42611	5906.827	5881.728	6188.384	3509.635	1665.486	4197.729	7394.17	91,052.23
	ET	2.987458	1.140728	2.762242	6.849603	3.566721	1.508286	1.301149	2.112364	1.546674	5.534626	3.041972	1.531272	1.76199
	Rank	1	2	12	13	3	9	6	5	8	10	7	4	11
${C}_{15}$	Mean	1420.829	1440.266	3294.843	5710.918	1569.994	3578.13	1546.269	1603.793	2446.452	1624.394	5948.724	3152.854	13,995.64
	Std	2.599676	12.39114	687.4668	1133.047	51.76615	2370.315	42.81284	305.9188	1898.234	54.4611	1504.696	2813.783	10,186.7
	ET	2.845627	1.206945	2.785167	6.874761	3.576565	1.517747	1.305058	2.015353	1.537448	5.567527	3.05451	1.553163	1.859887
	Rank	1	2	9	11	4	10	3	5	7	6	12	8	13
${C}_{16}$	Mean	1518.303	1540.237	7560.294	14,980.49	1659.677	7504.47	6651.267	1563.504	6211.694	1745.582	25,866.69	9674.444	4836.23
	Std	0.18902	7.627427	3842.977	13,122.67	17.88227	4781.062	5421.678	13.28443	1664.527	114.6471	12,793.27	5421.193	3312.171
	ET	2.761322	1.084223	2.716524	6.880903	3.401858	1.432233	1.260428	2.404585	1.466779	5.319318	3.003796	1.475542	1.672515
	Rank	1	2	10	12	4	9	8	3	7	5	13	11	6
${C}_{17}$	Mean	1620.33	1651.546	1897.385	2072.1	1794.952	2105.687	2000.377	1854.36	1758.913	1702.789	2133.828	1971.181	1839.25
	Std	0.413013	59.12325	88.70904	216.3476	42.53324	182.3609	162.1172	69.66636	94.10053	40.68201	158.7172	131.4634	60.70065
	ET	2.922317	1.104349	2.750624	6.929148	3.46053	1.466667	1.256555	2.35024	1.499318	5.430021	3.026472	1.504192	1.723349
	Rank	1	2	8	11	5	12	10	7	4	3	13	9	6
${C}_{18}$	Mean	1739.769	1759.38	1809.104	1849.202	1940.462	1831.594	1874.797	1875.756	1794.966	1783.928	1880.1	1777.377	1781.317
	Std	9.390864	19.15441	17.27001	12.63976	69.12122	12.20181	54.70165	88.60647	75.1563	10.82718	124.9458	6.216098	2.739942
	ET	3.422864	1.373381	2.961349	7.028175	3.942321	1.707681	1.543518	2.528669	1.731379	6.260958	3.26785	1.687528	1.957027
	Rank	1	2	7	9	13	8	10	11	6	5	12	3	4
${C}_{19}$	Mean	1822.393	1843.219	1,555,684	6,182,483	1980.379	12,953.85	25,158.44	22,596.97	21,467.39	31,882.7	10,407.72	23,605.32	13,773.29
	Std	0.654604	10.52135	2,046,315	8,174,037	64.62466	3980.99	15,769.55	12,776.37	15,003.12	6443.527	2530.639	21,206.51	7131.201
	ET	2.914595	1.113373	2.765405	6.883858	3.491219	1.497679	1.288268	2.305685	1.562607	5.506185	3.025709	1.527084	1.736712
	Rank	1	2	12	13	3	5	10	8	7	11	4	9	6
${C}_{20}$	Mean	1923.272	1928.873	226,966.5	763,650	2131.778	136,058.9	37,628.15	1938.779	5703.18	4957.527	43,724.1	26,929.86	6569.977
	Std	0.369818	4.100448	196,422.4	717,757.5	29.81473	154,801.6	24,971.29	7.622458	6158.53	5637.36	23,097.21	37,993.29	3433.467
	ET	5.681011	2.198231	3.872663	7.996336	5.715268	2.626269	2.383587	3.433651	2.66522	8.937754	4.250877	2.666788	2.843785
	Rank	1	2	12	13	4	11	9	3	6	5	10	8	7
${C}_{21}$	Mean	2067.076	2067.046	2197.937	2265.97	2031.928	2248.994	2248.143	2175.404	2208.342	2102.103	2299.254	2207.326	2078.461
	Std	12.47033	23.77396	41.12687	60.83282	9.028956	98.45771	98.31852	89.32691	56.29458	9.756751	83.94788	30.1848	11.08937
	ET	3.495783	1.364452	3.013562	7.143803	3.98272	1.719226	1.574374	2.453979	1.822637	6.275645	3.285749	1.73905	1.986589
	Rank	3	2	7	12	1	11	10	6	9	5	13	8	4
${C}_{22}$	Mean	2226.4	2284.466	2324.208	2299.285	2458.014	2362.314	2345.687	2284.106	2349.418	2334.645	2409.16	2355.396	2332.995
	Std	1.12E-05	64.10826	16.92359	32.51713	90.23298	76.53896	67.04678	66.63367	4.098373	69.9699	15.79384	8.338653	52.5004
	ET	3.450832	1.401169	2.995836	7.09781	3.970771	1.713638	1.508972	2.28657	1.765494	6.173443	3.298679	1.661932	1.972283
	Rank	1	3	5	4	13	11	8	2	9	7	12	10	6
${C}_{23}$	Mean	2347.789	2331.702	2611.25	2996.763	2327.955	2777.291	2353.455	2312.14	2336.94	2348.862	2327.6	2342.014	2347.076
	Std	32.62054	1.802696	88.39054	165.7393	0.319001	229.1817	5.979814	40.81921	10.56695	8.954942	4.84E-11	23.3774	3.410703
	ET	3.98756	1.527526	3.143476	7.226233	4.214015	1.932928	1.632912	2.323814	1.882882	6.522159	3.433647	1.846398	2.072976
	Rank	8	4	11	13	3	12	10	1	5	9	2	6	7
${C}_{24}$	Mean	2640.107	2660.734	2703.659	2740.655	2696.217	2765.507	2684.208	2697.026	2776.933	2677.491	2839.94	2679.387	2692.296
	Std	1.129135	10.68091	22.13161	35.48547	107.6211	65.64839	22.30794	78.50251	174.1397	9.670808	104.13	9.485398	14.66617
	ET	4.261043	1.473416	3.139665	7.218091	4.388387	1.931915	1.671435	2.36283	1.931631	6.710749	3.452823	1.886706	2.105212
	Rank	1	2	9	10	7	11	5	8	12	3	13	4	6
${C}_{25}$	Mean	2530	2659.97	2764.229	2900.871	2743.203	2703.2	2803.692	2719.557	2790.813	2798.492	2832.176	2809.082	2762.881
	Std	6.57E-05	149.894	59.07219	41.23084	152.7406	166.5024	19.6523	126.547	18.09781	3.569194	100.1059	14.21019	140.1599
	ET	4.214127	1.531454	3.208993	7.275993	4.411459	1.908476	1.737936	2.390203	2.116833	6.843712	3.484969	1.996938	2.187695
	Rank	1	2	7	13	5	3	10	4	8	9	12	11	6
${C}_{26}$	Mean	2939.972	2957.344	3115.494	3342.127	2932.516	3186.417	2940.533	2956.37	2974.419	2968.799	2956.556	2957.712	2989.152
	Std	77.54716	27.48258	116.453	66.54141	1.89E-08	385.9018	104.3606	27.47506	13.34528	20.98996	25.60616	27.44166	9.921065
	ET	3.678597	1.413734	3.093918	7.17875	4.198947	1.909976	1.591243	2.284097	1.870087	6.480475	3.367676	1.786583	2.05537
	Rank	2	6	11	13	1	12	3	4	9	8	5	7	10
${C}_{27}$	Mean	2862.955	2948.384	3467.513	3866.638	2909.502	3719.161	3242.777	2934.961	3332.336	3268.472	3981.274	2939.218	2931.771
	Std	96.9765	91.33146	162.0792	310.1073	50.59636	598.8449	317.2958	0.038934	469.9391	488.6216	777.5515	89.98807	221.6715
	ET	4.780488	1.621275	3.294333	7.413718	4.558512	2.093386	1.800535	2.440357	2.04527	7.057821	3.533837	1.97621	2.317277
	Rank	1	6	10	12	2	11	7	4	9	8	13	5	3
${C}_{28}$	Mean	3126.15	3145.346	3202.102	3280.69	3375.87	3224.543	3241.297	3128.888	3155.531	3154.422	3275.144	3177.256	3203.298
	Std	0.15309	10.0279	25.45743	142.6992	96.13972	58.84485	12.56083	2.689293	44.05881	40.76261	16.32711	39.49311	45.82105
	ET	4.966231	1.564901	3.300359	7.489424	4.652527	2.036375	1.848247	2.489605	2.105183	7.322945	3.630412	2.008709	2.266859
	Rank	1	3	7	12	13	9	10	2	5	4	11	6	8
${C}_{29}$	Mean	3199.763	3244.848	3547.719	3875.438	3137.2	3665.733	3340.237	3287.983	3403.426	3381.846	3518.427	3360.736	3296.269
	Std	20.83825	126.6863	76.36163	71.49689	7.51E-05	215.8928	133.0303	174.2557	109.7254	91.61967	15.96006	105.1771	194.2335
	ET	4.246133	1.48251	3.205908	7.422436	4.429292	1.928158	1.74264	2.408991	1.988376	6.931333	3.541678	1.885937	2.195382
	Rank	2	3	11	13	1	12	6	4	9	8	10	7	5
${C}_{30}$	Mean	3242.31	3218.034	3313.113	3434.28	3175.212	3283.043	3405.644	3246.523	3314.547	3257.359	3402.368	3315.496	3284.129
	Std	13.50853	18.88143	44.05249	77.70265	3.260145	62.57449	118.6531	66.40669	98.04684	35.42945	210.7321	89.40796	44.90439
	ET	4.240703	1.678074	3.287334	7.449646	4.542081	2.003263	1.783916	2.439172	2.014811	7.115835	3.578222	1.984954	2.229906
	Rank	3	2	8	13	1	6	12	4	9	5	11	10	7
Sum rank		52	89	278	347	138	294	246	129	206	203	262	192	203
Mean rank		1.793103	3.068966	9.586207	11.96552	4.758621	10.13793	8.482759	4.448276	7.103448	7	9.034483	6.62069	7
Total rank		1	2	10	12	4	11	8	3	7	6	9	5	6

## CBOA for real world applications

In this section, we will show the effectiveness of CBOA in solving real-world problems. To this end, CBOA and competing algorithms are used in the optimization of four engineering applications: (i) pressure vessel design (PVD), (ii) speed reducer design (SRD), (iii) welded beam design (WBD), and (iv) structural tension/compression springs (TCSD). Mathematical models, details, and information about these technical challenges are expressed for PVD in Ref.^34^, for SRD in Refs.^35,36^, and for WBD and TCSD in Ref.^16^. The optimization results of these four engineering optimization problems are published in Table 12. Based on the analysis of the results, it is clear that the CBOA approach is the first best optimizer in solving all four studied problems compared to competing algorithms.

**Table 12:** Assessment results of engineering optimization applications.

		CBOA	CMA	DE	HBA	MPA	TSA	WOA	MVO	GWO	TLBO	GSA	PSO	GA
PVD	Mean	5882.405	5884.773	6041.266	6120.411	5891.389	5895.471	6069.081	6481.645	6331	6845.126	6268.202	6648.439	6429.323
	Best	5883.117	5884.772	6033.975	6112.525	5916.011	5919.822	5921.851	6042.598	6169.107	11,610.02	5920.786	6583.892	6392.769
	Std	23.71331	31.13375	31.20048	38.25817	28.94939	13.91933	66.66323	327.2262	126.6938	5794.505	496.4605	657.9637	351.4736
	Median	5886.142	5887.641	6039.357	6118.226	5890.172	5894.597	6420.413	6401.767	6322.551	6842.214	6116.786	7591.092	6904.266
	ET	1.123067	1.741255	1.602954	3.711218	2.214646	0.964079	0.87132	1.204496	0.994188	3.465667	1.770839	0.942969	1.097691
	Rank	1	2	5	7	3	4	6	11	9	13	8	12	10
SRD	Mean	2999.639	3000.889	3000.081	3001.774	3011.639	3003.451	3009.664	3109.197	3032.689	3069.812	3174.361	3190.57	3299.515
	Best	2996.001	2996.084	2996.082	2996.126	3004.747	3001.46	3004.2	3008.679	3005.841	3033.503	3054.081	3070.537	3031.941
	Std	1.623508	4.163184	2.014972	5.218941	10.36777	1.934384	5.845356	79.73926	13.03514	18.09716	92.6902	17.14034	57.09594
	Median	2998.672	3000.33	2999.746	3000.341	3010.25	3002.997	3008.336	3109.197	3030.877	3069.503	3160.762	3202.25	3292.835
	ET	1.090244	0.810638	1.647167	3.706859	2.279256	1.046693	0.900268	1.220179	1.022635	3.528911	1.766919	0.988105	1.158639
	Rank	1	3	2	4	7	5	6	10	8	9	11	12	13
WBD	Mean	1.696107	1.726626	1.726898	1.703194	1.891812	1.728636	1.729938	2.233938	1.732494	1.820613	2.548378	2.122687	1.765745
	Best	1.724628	1.724686	1.724629	1.672383	1.865877	1.727431	1.728767	1.822263	1.727242	1.760978	2.175088	1.875894	1.838134
	Std	0.004327	0.007131	0.005122	0.017423	0.007959	0.000287	0.001159	0.325054	0.004874	0.027587	0.256276	0.034876	0.139712
	Median	1.725382	1.72563	1.7256	1.726194	1.883295	1.728595	1.729897	2.248315	1.73023	1.823089	2.499173	2.10046	1.938897
	ET	1.125416	0.700585	1.497918	3.548905	1.985386	0.856819	0.754486	1.143285	0.882747	3.086722	1.64934	0.830966	0.986142
	Rank	1	3	4	2	10	5	6	12	7	9	13	11	8
TCSD	Mean	0.012685	0.012715	0.012696	0.01279	0.013888	0.012793	0.012806	0.014945	0.014588	0.01295	0.013554	0.014156	0.013182
	Best	0.012663	0.012705	0.012664	0.012758	0.013208	0.012776	0.01278	0.013299	0.01292	0.012812	0.012977	0.013141	0.012879
	Std	0.001022	0.006146	0.001566	0.007412	0.006136	0.005667	0.004189	0.002292	0.001636	0.007825	0.000289	0.002091	0.000378
	Med	0.012682	0.01271	0.01269	0.01278	0.013766	0.012796	0.012809	0.013306	0.014141	0.012955	0.013482	0.013113	0.013063
	ET	1.191738	0.828262	2.062968	5.196861	2.595398	1.102364	0.942416	1.76268	1.124489	4.072516	2.269854	1.128144	1.292512
	Rank	1	3	2	4	10	5	6	13	12	7	9	11	8

## Conclusions and future works

This paper introduced a new human-based metaheuristic algorithm called the chef-based optimization algorithm (CBOA) and designed it to address optimization issues. The process of learning cooking skills by people who attend training cooking courses inspired the implementation of the proposed CBOA. Different phases of the cooking training process were mathematically modeled to design the CBOA implementation. The CBOA’s performance was evaluated on fifty-two benchmark functions, including seven unimodal functions, six high-dimensional multimodal functions, ten fixed-dimensional multimodal functions, and 29 functions of the CEC 2017 test suite The optimization results showed that CBOA could be used effectively in solving optimization problems due to its ability to maintain a balance between exploration and exploitation. Moreover, the simulation results showed that CBOA is more efficient and competitive than the twelve compared algorithms because it usually provides better solutions.

In addition, the employment of the CBOA on four engineering optimization issues demonstrated the high ability of the proposed approach to address real-world applications.

The proposed CBOA algorithm is a stochastic approach and therefore has some shortages and limitations. As with all metaheuristic algorithms, there is no guarantee that the solutions obtained from the CBOA for optimization problems are equal to the global optima of those problems. Although the CBOA has provided reasonable solutions to most of the objective functions studied in this paper, according to the NFL theorem, there are no preconditions for its successful implementation in all optimization applications Therefore, of course, there is a shortage and limitation of the proposed CBOA that its application may fail in some optimization problems. Also, it is always possible that researchers will design newer metaheuristic algorithms to provide better solutions to real optimization problems than existing algorithms, such as the proposed CBOA method.

The introduction of the CBOA opens research directions and tasks for future work. The most specific research potential for the CBOA is the development of binary and multi-objective versions of this proposed approach. The employment of CBOA in optimization applications in various sciences and real-world challenges are other proposals in this paper.

## Data Availability

All data generated or analyzed during this study are included directly in the text of this submitted manuscript. There are no additional external files with datasets.

## Code availability

The source code of the CBOA is available at: https://uk.mathworks.com/matlabcentral/fileexchange/114480-chef-based-optimization-algorithm-cboa.
